# Multi-omics Analysis Reveals How Intratumoral Bacteria Shape the Immune Microenvironment in Gastric Cancer

**DOI:** 10.1093/gpbjnl/qzaf132

**Published:** 2025-12-27

**Authors:** Yang Mi, Die Dai, Xia Xue, Haiming Qin, Feifei Ren, Barry J Marshall, Alfred Tay, Ihtisham Bukhari, Xiaojie Li, Shaogong Zhu, Yong Yu, Wanqing Wu, Yan Tan, Youcai Tang, Xin Xie, Haiqing Bai, Xiaochen Yin, Pengyuan Zheng

**Affiliations:** Henan Key Laboratory for Helicobacter pylori and Digestive Tract Microecology, The Fifth Affiliated Hospital of Zhengzhou University, Zhengzhou 450052, China; Institute of Rehabilitation Medicine, Henan Academy of Innovations in Medical Science, Zhengzhou 450046, China; Tianjian Laboratory of Advanced Biomedical Sciences, Zhengzhou University, Zhengzhou 450000, China; Department of Gastroenterology, The Fifth Affiliated Hospital of Zhengzhou University, Zhengzhou 450052, China; Shenzhen Xbiome Biotechnology Co., Ltd., Shenzhen 518000, China; Henan Key Laboratory for Helicobacter pylori and Digestive Tract Microecology, The Fifth Affiliated Hospital of Zhengzhou University, Zhengzhou 450052, China; Institute of Rehabilitation Medicine, Henan Academy of Innovations in Medical Science, Zhengzhou 450046, China; Tianjian Laboratory of Advanced Biomedical Sciences, Zhengzhou University, Zhengzhou 450000, China; Henan Key Laboratory for Helicobacter pylori and Digestive Tract Microecology, The Fifth Affiliated Hospital of Zhengzhou University, Zhengzhou 450052, China; Institute of Rehabilitation Medicine, Henan Academy of Innovations in Medical Science, Zhengzhou 450046, China; Tianjian Laboratory of Advanced Biomedical Sciences, Zhengzhou University, Zhengzhou 450000, China; Henan Key Laboratory for Helicobacter pylori and Digestive Tract Microecology, The Fifth Affiliated Hospital of Zhengzhou University, Zhengzhou 450052, China; Institute of Rehabilitation Medicine, Henan Academy of Innovations in Medical Science, Zhengzhou 450046, China; Tianjian Laboratory of Advanced Biomedical Sciences, Zhengzhou University, Zhengzhou 450000, China; Helicobacter pylori Research Laboratory, Marshall Centre for Infectious Diseases Research and Training, School of Biomedical Sciences, University of Western Australia, Perth, WA 6009, Australia; Helicobacter pylori Research Laboratory, Marshall Centre for Infectious Diseases Research and Training, School of Biomedical Sciences, University of Western Australia, Perth, WA 6009, Australia; Henan Key Laboratory for Helicobacter pylori and Digestive Tract Microecology, The Fifth Affiliated Hospital of Zhengzhou University, Zhengzhou 450052, China; Institute of Rehabilitation Medicine, Henan Academy of Innovations in Medical Science, Zhengzhou 450046, China; Tianjian Laboratory of Advanced Biomedical Sciences, Zhengzhou University, Zhengzhou 450000, China; Henan Key Laboratory for Helicobacter pylori and Digestive Tract Microecology, The Fifth Affiliated Hospital of Zhengzhou University, Zhengzhou 450052, China; Institute of Rehabilitation Medicine, Henan Academy of Innovations in Medical Science, Zhengzhou 450046, China; Tianjian Laboratory of Advanced Biomedical Sciences, Zhengzhou University, Zhengzhou 450000, China; Department of Gastrointestinal Surgery, People’s Hospital of Zhengzhou University, Zhengzhou 450003, China; Henan Key Laboratory for Helicobacter pylori and Digestive Tract Microecology, The Fifth Affiliated Hospital of Zhengzhou University, Zhengzhou 450052, China; Institute of Rehabilitation Medicine, Henan Academy of Innovations in Medical Science, Zhengzhou 450046, China; Tianjian Laboratory of Advanced Biomedical Sciences, Zhengzhou University, Zhengzhou 450000, China; Department of Gastroenterology, The Fifth Affiliated Hospital of Zhengzhou University, Zhengzhou 450052, China; Department of Gastrointestinal Surgery, The Fifth Affiliated Hospital of Zhengzhou University, Zhengzhou 450052, China; Shenzhen Xbiome Biotechnology Co., Ltd., Shenzhen 518000, China; Department of Pediatrics, The Fifth Affiliated Hospital of Zhengzhou University, Zhengzhou 450052, China; Henan Academy of Innovations in Medical Science, Zhengzhou 451100, China; Xellar Biosystems, Boston, MA 02142, USA; Henan Academy of Innovations in Medical Science, Zhengzhou 451100, China; Xellar Biosystems, Boston, MA 02142, USA; Shenzhen Xbiome Biotechnology Co., Ltd., Shenzhen 518000, China; Henan Key Laboratory for Helicobacter pylori and Digestive Tract Microecology, The Fifth Affiliated Hospital of Zhengzhou University, Zhengzhou 450052, China; Institute of Rehabilitation Medicine, Henan Academy of Innovations in Medical Science, Zhengzhou 450046, China; Tianjian Laboratory of Advanced Biomedical Sciences, Zhengzhou University, Zhengzhou 450000, China; Department of Gastroenterology, The Fifth Affiliated Hospital of Zhengzhou University, Zhengzhou 450052, China

**Keywords:** Tumor microenvironment, Gastric cancer, Immune-related protein, Spatial bacterial community, Tryptophan metabolism

## Abstract

The occurrence and progression of gastric cancer (GC) are closely associated with dysbiosis of the gastric microbiota and alteration in host microenvironments. However, the interaction between intratumoral bacteria and gastric microenvironments remains incompletely understood. In this study, we characterized the biological profiles of intratumoral bacteria, metabolome, and proteome in 20 GC tumors and paired non-tumor tissues, in combination with 6 independent datasets (comprising 477 gastric tissue biopsies and 534 normal tissues), as well as mucosal tissues from 10 individuals without GC. We found that the diversity and richness of gastric microbiota were significantly higher in tumor tissues than in non-tumor tissues. In contrast, the lowest biodiversity, at both the genus and species levels, was found in the microbiota of individuals without GC. Specifically, tumors were enriched with *Bacteroides thetaiotaomicron*, *Lactobacillus parabrevis*, *Brevundimonas nasdae*, and *Brevundimonas vesicularis*. We also identified 39 human immunity-related proteins, particularly in the tryptophan metabolic pathway, which were differentially expressed across various microenvironments (tumor and non-tumor). Furthermore, we found that several pathways involved in the human immune system and associated with the gastric microbiota, such as thiazole biosynthesis II, pyrimidine deoxyribonucleoside salvage, superpathway of pyrimidine deoxyribonucleoside salvage, and superpathway of heme biosynthesis from uroporphyrinogen-III, hold potential as biomarkers for early detection of GC. Our results provide a comprehensive framework for investigating the complex interactions between the tumor immune microenvironment and intratumoral bacterial community.

## Graphical abstract

**Figure:**
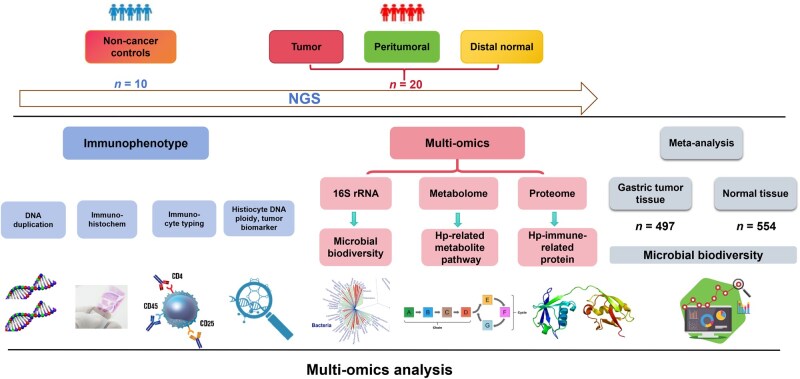

## Introduction

Pathogenic bacteria are considered oncopromoters in various cancers, including gastric cancer (GC) [1]. A strong association has been found between specific bacteria within tumor tissues (intratumoral bacteria) and carcinogenesis [2,3]. These intratumoral bacteria can influence tumor development and progression by contributing to chronic inflammation, genotoxicity, metabolic alterations, and disruption of cellular integrity in the host. Reduced immune surveillance and increased nutrient release in tumors provide a more favorable niche for bacterial proliferation [4,5]. Although the origins of intratumoral bacteria remain controversial across tumor types, it has been established that each tumor microenvironment harbors distinct bacterial communities, which may serve as potential biomarkers of tumorigenesis and individual heterogeneity [1,6]. Therefore, a better understanding of the tumor microenvironment and the functions of intratumoral bacteria is crucial for developing effective therapeutics and metabolically engineered microorganisms against cancer. However, the relatively lower biomass of intratumoral bacteria compared to host tissues makes it challenging to characterize their biological signatures across different tumor environments.

GC is a multifactorial disease influenced by genetics, environment, dietary habits, immune function, and chronic gastric disease [7–10]. Despite advances in modern medicine, GC remains a leading cause of cancer-related deaths worldwide (WHO, 2025). The 5-year survival rate for GC is approximately 25%, which drops to under 5% once the tumors spread to the peritoneum [11,12]. Gastric carcinogenesis has been shown to positively correlate with the presence of intragastric bacteria and their metabolites [13]. *Helicobacter pylori* (Hp) is one of the first bacterial species confirmed as the oncogenic agent for gastric adenocarcinoma [14,15]. Chronic Hp infection develops into chronic gastritis, intestinal metaplasia, dysplasia, and eventually GC [16]. During this progression, Hp triggers gastric carcinogenesis pathways by altering mucosal functions at both metabolic and immune levels [8]. In addition, Hp releases various virulence factors to increase the levels of pro-inflammatory cytokines and chemokines, ultimately leading to a chronic inflammatory response in the gastric mucosa [17,18]. Hp infection also triggers the infiltration of immune cells into the gastric mucosa and release of cytokines such as interleukin-1 (IL-1), tumor necrosis factor-alpha (TNF-α), and interferon-gamma (IFN-γ) [19]. By regulating the expression of surface molecules, Hp can evade recognition by regulatory T cells (Tregs) and other immunosuppressive cells, building a favorable microenvironment for bacterial persistence and tumor progression [20,21]. Conversely, gastric bacterial infection simulates the release of pro-inflammatory cytokines, contributing to DNA damage and carcinogenesis [22,23]. Therefore, a comprehensive characterization of intratumoral bacteria and their roles in regulating host immune responses could fill the gaps in establishing personalized medicine and improving the prognosis of GC patients.

Recent cohort studies have reported that higher bacterial diversity and richness were found in non-tumor tissues from GC patients than in malignant tumor tissues [24,25]. Moreover, bacterial communities vary substantially between individuals owing to the specific immune microenvironments of each patient [24–26]. It is highly heterogeneous to study the interaction between gastric bacteria and tumor microenvironment both temporally and spatially, particularly without controlling for genetic background and other personal factors. The composition and status of the intragastric microbiome have been linked to the progression of GC, and the intragastric microbiome can regulate immune responses in GC patients by influencing host immune-related gene expression and metabolic activities [27]. Metabolomic analyses, such as proton nuclear magnetic resonance (^1^H NMR), liquid chromatography-mass spectrometry (LC-MS), gas chromatography-mass spectrometry (GC-MS), ultra-performance liquid chromatography-mass spectrometry (UPLC-MS), and liquid chromatography-time-of-flight mass spectrometry (LC-TOF-MS), have shown that metabolic changes in tumors can serve as biomarkers for early diagnosis of gastric carcinogenesis [25,28]. These biomarkers are involved in different metabolic pathways, including energy metabolism (glucose, lactic acid, succinic acid, *etc.*), amino acid metabolism (histidine, alanine, threonine, *etc.*), lipid metabolism (cholesterol and choline), nucleic acid metabolism (adenine, xanthine nucleoside, inosine, *etc.*). Intestinal microbial metabolites, such as branched-chain amino acids (BCAAs) and trimethylamine-*N*-oxide (TMAO), have been linked to the abundance and functional activity of specific intragastric bacterial species [29,30]. In addition to the intragastric bacterial community, the dynamics of protein expression under different microenvironments play a crucial role in mediating pathogen-host interactions, which are key drivers of gastric carcinogenesis pathways [25]. Despite these insights, comprehensive multi-omics studies of intratumoral bacteria in GC remain limited, thereby constraining advances in GC treatment.

Meta-genomics, metabolomics, and proteomics are cutting-edge integrative strategies that can be employed to reveal the multidimensional profiles of gastric intratumoral bacteria and the heterogeneous tumor microenvironment. We herein characterized the bacterial community, metabolic profile, and functional roles of intratumoral bacteria in GC patients by performing full-length 16S rRNA sequencing and metabolomic and proteomic measurements on paired GC and non-tumor tissues (peritumoral and distal normal). Moreover, a meta-analysis based on 6 independent datasets comprising 1011 samples was conducted to validate the network of intragastric bacteria, metabolites, and functional pathways involved in gastric carcinogenesis. This work aims to characterize multiple features of intratumoral bacteria across different tumor microenvironments, thereby improving our understanding of microbial-driven carcinogenesis in GC.

## Results

### Composition of gastric bacteria in GC and matched non-tumor tissues

**Figure 1:**
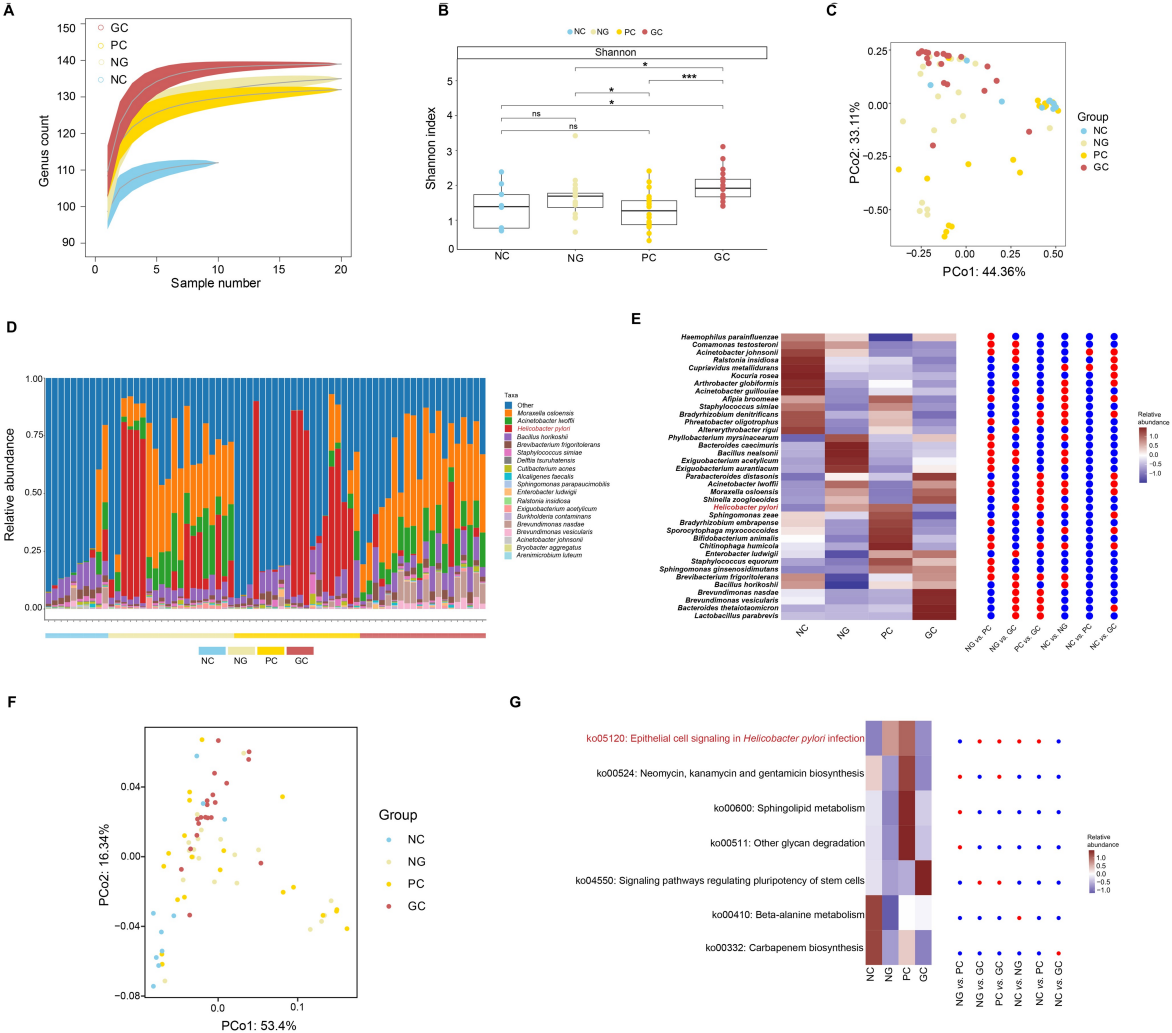
Altered intratumoral bacteria in four groups **A**. Rarefaction curves illustrating the number of bacterial genera passing all filters per the number of analyzed samples. Light shading represents confidence intervals (0.95) estimated via 100 random sub-samplings. **B**. Bacterial diversity among groups. The diversity was evaluated by Shannon indices, with the significance at the species level assessed by the Wilcoxon test (*, *P* < 0.05; ***, *P* < 0.001; ns, not significant). **C**. PCoA plot of species abundance based on Bray–Curtis dissimilarity. The x- and y-axes represent the first and second principal coordinates, with the percentage of variance indicated in each dimension. **D**. Phylogenetic composition of the top 20 microbial taxa at the species level, ordered by groups (NC, NG, PC, and GC). **E**. Heatmap of differentially abundant bacteria between groups. Each tile represents the scaled relative abundance of taxa. The red dots in the right panel denote bacteria that are significantly different between groups, based on MaAsLin2 analysis (*q* < 0.3) and two-proportions Z-test (*P* < 0.05), whereas the blue dots denote bacteria that are not significantly different between groups. **F**. PCoA plot of predicted bacterial metabolic pathway abundance based on Bray–Curtis dissimilarity. **G**. Heatmap of differentially expressed gastric bacterial KEGG pathways between groups. Each tile represents the scaled relative abundance of pathways. The red dots on the right panel denote significantly differential functional pathways between groups (LEfSe LDA > 2.5; MaAsLin2 *q* < 0.2; |log_2_ FC| > 2), whereas the blue dots denote functional pathways that are not significantly different between groups. PCoA, principal coordinate analysis; PCo, principal coordinate; NC, non-cancer control; NG, normal gastric mucosa; PC, para-carcinoma; GC, gastric cancer; KEGG, Kyoto Encyclopedia of Genes and Genomes; FC, fold change; LEfSe, Linear discriminant analysis Effect Size; LDA, linear discriminant analysis.

To explore the composition of intratumoral bacteria across different gastric tumor microenvironments, we performed full-length 16S rRNA gene amplicon sequencing of GC tissues, para-carcinoma (PC) tissues, and adjacent normal gastric mucosa (NG) tissues from 20 GC patients (Table S1). Additionally, gastric mucosal tissue samples of 10 individuals without GC were included as non-cancer controls (NCs). We found a higher bacterial load in GC samples than in non-tumor tissues (PC, NG, and NC) (**Figure 1**A). The Shannon index, which measures microbial species richness and evenness, was significantly higher in GC than in PC, NG, and NC (*P* < 0.05, Wilcoxon test; Figure 1B). Beta diversity was analyzed using principal coordinate analysis (PCoA) with Bray–Curtis dissimilarity to compare bacterial composition among groups. Notably, a Permutational Multivariate Analysis of Variance (PERMANOVA) revealed significant differences in species-level composition among the groups (*P* = 0.001), which were attributable to centroid displacements rather than differences in multivariate dispersions (beta dispersion, *P* = 0.21), as visualized in Figure 1C. In addition, the composition of bacterial communities differed among GC, PC, NG, and NC groups (Figure 1D, species level; Figure S1, genus level). Furthermore, we performed differential analysis between the groups. Hp was significantly enriched in PC and NG compared to GC and NC (Figure 1E, Figure S2), whereas *Lactobacillus*, *Lactococcus*, *Lactobacillus parabrevis*, *Bacteroides thetaiotaomicron*, *Brevundimonas vesicularis, Brevundimonas nasdae*, *Acinetobacter Iwoffii*, and *Moraxella osloensis* were significantly enriched in GC (two-proportions Z-test, *P* < 0.05; MaAsLin2, *q* < 0.3).

To identify the metabolic pathways associated with GC, we performed functional predictions using Phylogenetic Investigation of Communities by Reconstruction of Unobserved States (PICRUSt2) [31] with Kyoto Encyclopedia of Genes and Genomes (KEGG) as the reference database. A trend of separation among the groups (GC, NG, PC, and NC) was observed in the PCoA plot based on predicted bacterial metabolic pathway abundance (Figure 1F). This visual pattern was supported by a significant PERMANOVA result (*P* = 0.001), although the concurrent significance in multivariate dispersion (beta dispersion, *P* = 0.01) suggests that the group differences may be influenced by heterogeneity in within-group variations. Differential analysis [LDA Effect Size (LEfSe), linear discriminant analysis (LDA) > 2.5; MaAsLin2, *q* < 0.2; |log_2_ FC| > 2] between the groups identified seven differential pathways accordingly (Figure 1G). Notably, a pathway involved in “epithelial cell signaling in Hp infection” was upregulated in PC and NG compared to GC, while the pathways of “ko00410: beta-alanine metabolism” and “ko00332: carbapenem biosynthesis” were upregulated in the NC group.

### Meta-analysis of GC-associated microbiota based on public databases

To characterize consistent alterations in gastric bacterial communities between GC tissues and non-tumor tissues across public datasets for validation, we performed meta-analysis on seven datasets, including six publicly available 16S rRNA sequencing datasets and one generated in the current study (**Table 1**). The meta-analysis included 497 gastric tumor samples and 554 non-tumor ones. Since studies varied across multiple biological and technical factors (*e.g.*, sampling sites and DNA extraction methods), we evaluated the effect of study-associated heterogeneity on gastric microbial composition. The microbial alpha diversity was significantly increased in gastric tumor compared with non-tumor tissues in three datasets P1, P3, and P4, with the exception of P2 which showed an opposite trend (Figure S3A), while the remaining datasets showed non-significant differences, resulting in a highly significant global Analysis of Variance (ANOVA; *F*-statistics: *P* < 2E−16) but a non-significant overall pairwise comparison (*P* = 0.19). The PCoA analysis of seven studies using Bray–Curtis dissimilarity (Figure S3B) revealed distinct microbial community structures. PCo1 and PCo2 accounted for 23.4% and 17.9% of the variance, respectively. Study-wise projections and tumor-associated separation along PCo1 and PCo2, shown by vertical and horizontal box plots, suggest an association between tumor status and microbial profiles.

**Table 1:** Tumor microbiome studies of gastric cancer included in this meta-analysis

Project	No. of controls (non-tumor)	No. of cases (tumor)	Ref.
P1: PRJNA310127	157	134	[32]
P2: PRJNA428883	230	229	[33]
P3: NMDC10017675	36	37	[25]
P4: PRJEB21497	20	12	[34]
P5: PRJNA239281	10	11	[35]
P6: PRJNA413125	81	54	[36]
P7: ZhengDa	20	20	This study
Total	554	497	

**Figure 2:**
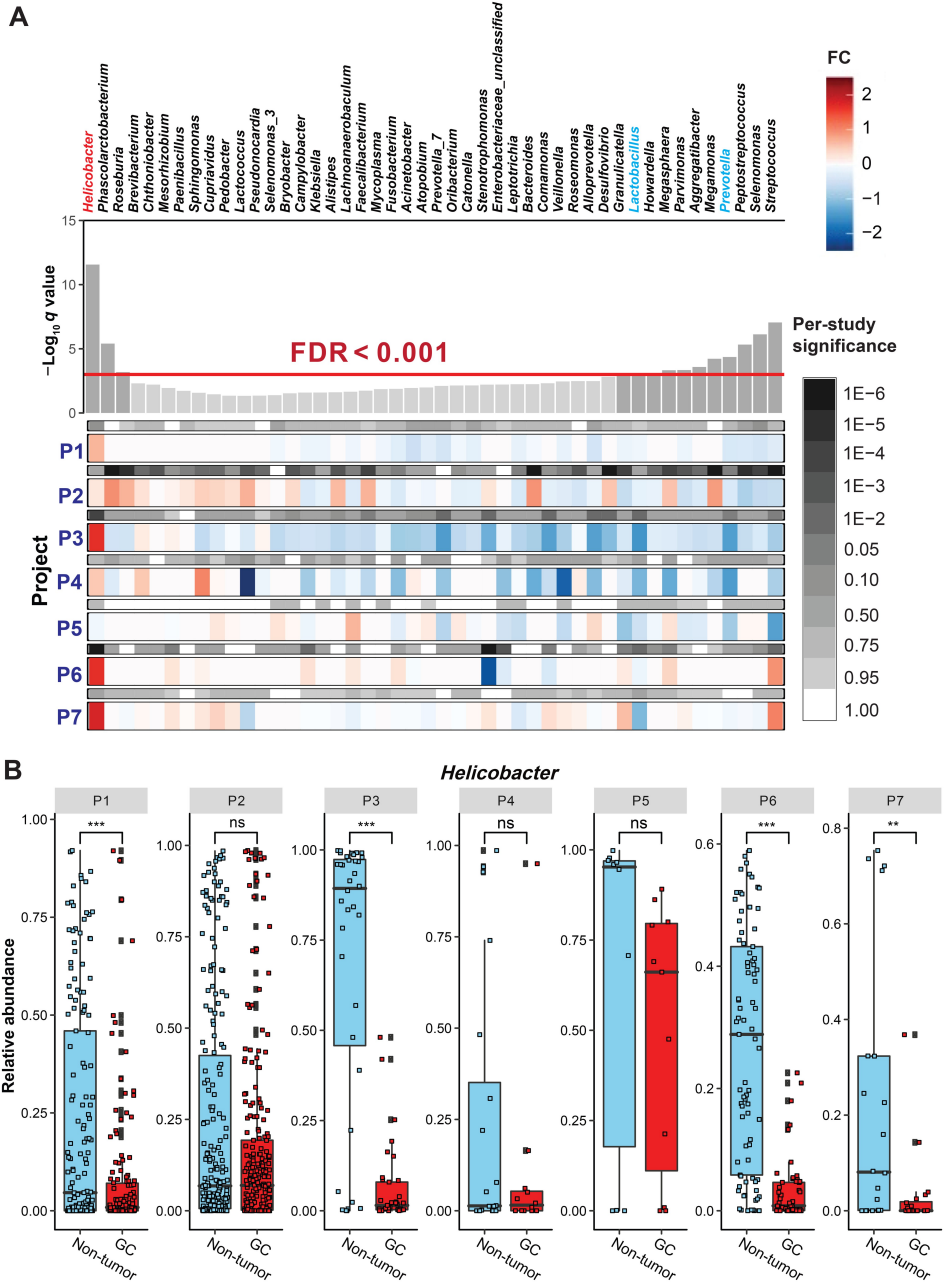
Meta-analysis of intragastric bacteria associated with GC **A**. Intragastric bacteria associated with GC. The bar height reflects the significance of the meta-analysis of intragastric bacteria based on blocked Wilcoxon tests. Underneath, genus-level importance was calculated with a two-sided Wilcoxon test (FDR-corrected *P*) in each study and displayed as gray heatmaps. The normalized FC within individual studies is color-coded. Red color denotes enrichment in non-tumor tissue, whereas blue color indicates enrichment within tumor tissue. Bacteria highlighted in red and blue represent taxa exhibiting significant changes, consistent with findings from our project (P7). **B**. Signature bacteria in tumor tissues compared to non-tumor tissues in GC patients. Gastric bacterial abundance from six independent studies (P1–P6) and the present study (P7) is plotted. Relative abundance of the bacterial genus in non-tumor tissues and tumor tissues is indicated in blue and red, respectively. The significance was assessed using a two-sided Wilcoxon test (**, *P* < 0.01; ***, *P* < 0.001; ns, not significant). FDR, false discovery rate.

To identify differential bacterial taxa in tumor tissues, we conducted a blocked (univariate) Wilcoxon test, treating “study” as a blocking factor to control confounding variables. Our meta-analysis [false discovery rate (FDR) < 0.001] identified 14 differentially abundant bacterial taxa between GC and non-tumor tissues (**Figure 2**A). Across all seven datasets, *Helicobacter* was enriched in non-tumor tissues (Figure 2B), whereas several beneficial bacteria, such as *Lactobacillus* and *Prevotella*, were enriched in GC tissues (Figure S5). In addition, the area under the receiver operating characteristic curve (AUROC) was calculated between GC and non-tumor groups, showing variable discrimination across taxa (meta-analysis, FDR = 0.001). AUROC values for most taxa were around 0.5 (Figure S4), while those for *Helicobacter* were high in several datasets (up to 0.9), indicating significant enrichment in non-tumor tissues. Overall, our meta-analysis indicates a consistent gastric bacterial composition pattern across different tissues and tumor microenvironments.

### Metabolomics analysis of tissues and multi-source non-tumor gastric tissues

**Figure 3:**
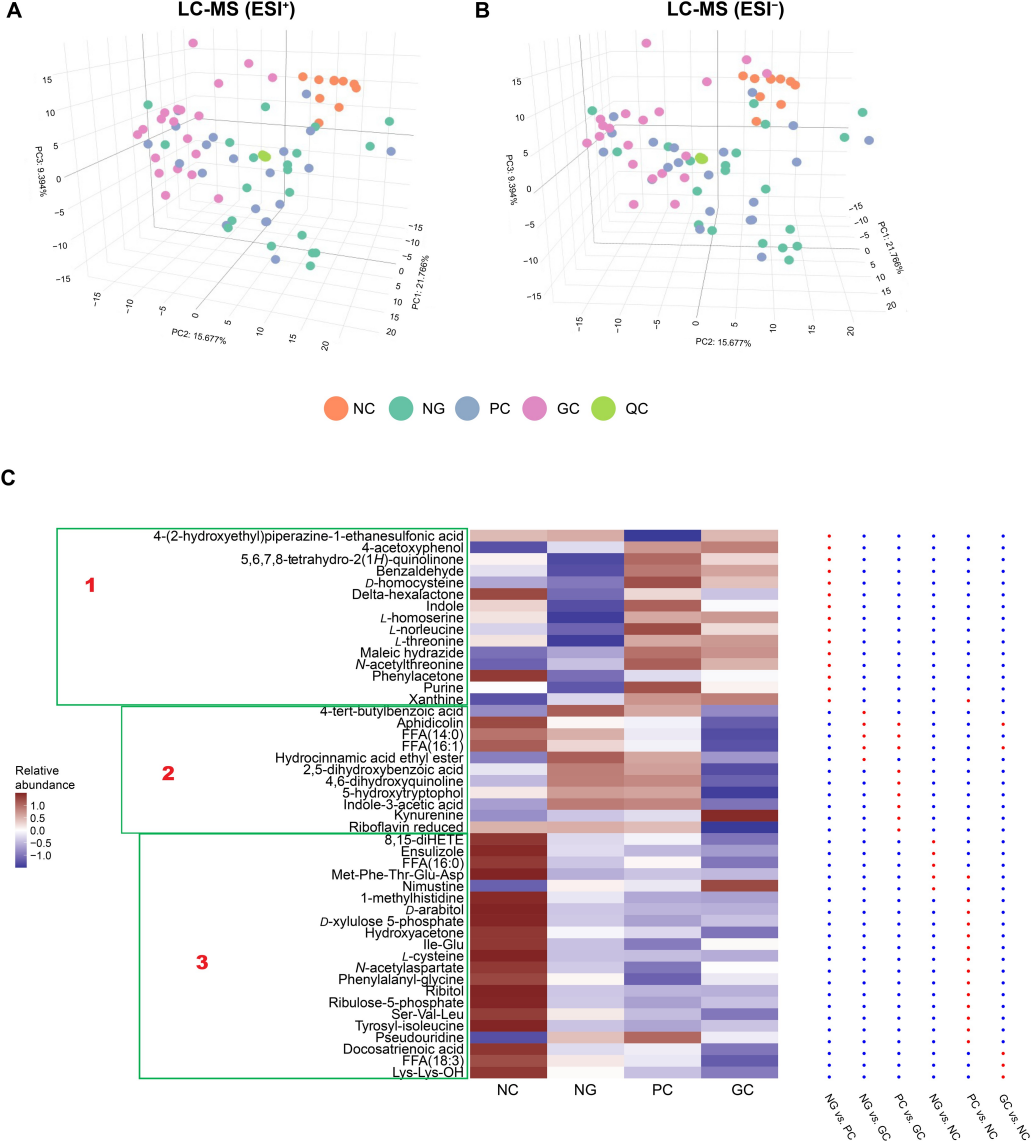
Metabolomic profiles across different sample groups **A**. PCA plot of metabolomic profiles detected by LC-MS (ESI^+^). **B**. PCA plot of metabolomic profiles detected by LC-MS (ESI^–^). QC samples, prepared by pooling equal aliquots from all samples, were used to evaluate instrument stability. **C**. Heatmap of differentially abundant metabolites across the NC, NG, PC, and GC groups. Each tile represents the scaled relative abundance of the metabolites. The heatmap is organized into three distinct clusters (labeled as Boxes 1, 2, and 3) based on the similarity of metabolite abundance patterns across groups: Box 1 is dominated by metabolites that drive differences between NG and PC, Box 2 features metabolites primarily distinguishing GC from NG and PC, and Box 3 is centered on metabolites that characterize differences between the NC group and the other groups (NG, PC, and GC). The red dots in the right panel denote metabolites that are significantly different between groups, based on sPLS-DA, whereas the blue dots denote metabolites that are not significantly different between groups. LC-MS, liquid chromatography-mass spectrometry; QC, quality control; PCA, principal component analysis; ESI, electrospray ionization; sPLS-DA, sparse partial least squares discriminant analysis.

Since the composition and functions of bacteria differ between GC tissues and multi-source non-tumor gastric tissues, which encompass paired PC tissues, adjacent NG tissues from GC patients, and gastric mucosal tissues derived from NCs, we analyzed the metabolite abundance to explore the role of gastric bacteria in GC-associated metabolic pathways. We performed untargeted metabolomics [32,33] analysis on tissues from each group and quantified 899 metabolites. Quality-control samples clustered tightly in the Principal Component Analysis (PCA) plot in both positive electrospray ionization (ESI^+^) and negative electrospray ionization (ESI^−^) modes (**Figure 3**A and B), indicating stable mass spectrometry (MS) performance and data reliability.

To identify gastric metabolites associated with GC, we performed differential analysis between the tumor and non-tumor groups using sparse partial least squares discriminant analysis (sPLS-DA). The differential metabolites were classified into three clusters based on the heatmap clustering pattern (Figure 3C). Notably, indole-3-acetic acid (IAA) and folic acid were grouped into the same cluster, which is related to immune response. The significantly different metabolites found between NG and PC were primarily related to the purine metabolism (sPLS-DA with *P* < 0.05; Box 1 in Figure 3C). In contrast, the significantly different metabolites found between GC and NG/PC (sPLS-DA with *P* < 0.05; Box 2 in Figure 3C) were annotated to the tryptophan metabolism pathway, including the synthesis of IAA, 5-hydroxytryptophol (5-HTOL), 4,6-dihydroxyquinoline, and kynurenine. The metabolic profile of the NC group was significantly different from that of the GC/NG/PC groups (sPLS-DA with *P* < 0.05; Box 3 in Figure 3C). Notably, the levels of kynurenine and nimustine were significantly higher (sPLS-DA with *P* < 0.05) in GC tissues, while other metabolites were more abundant in NG, PC, and NC tissues.

### Altered gastric proteome in GC tissues compared with non-tumor tissues

**Figure 4:**
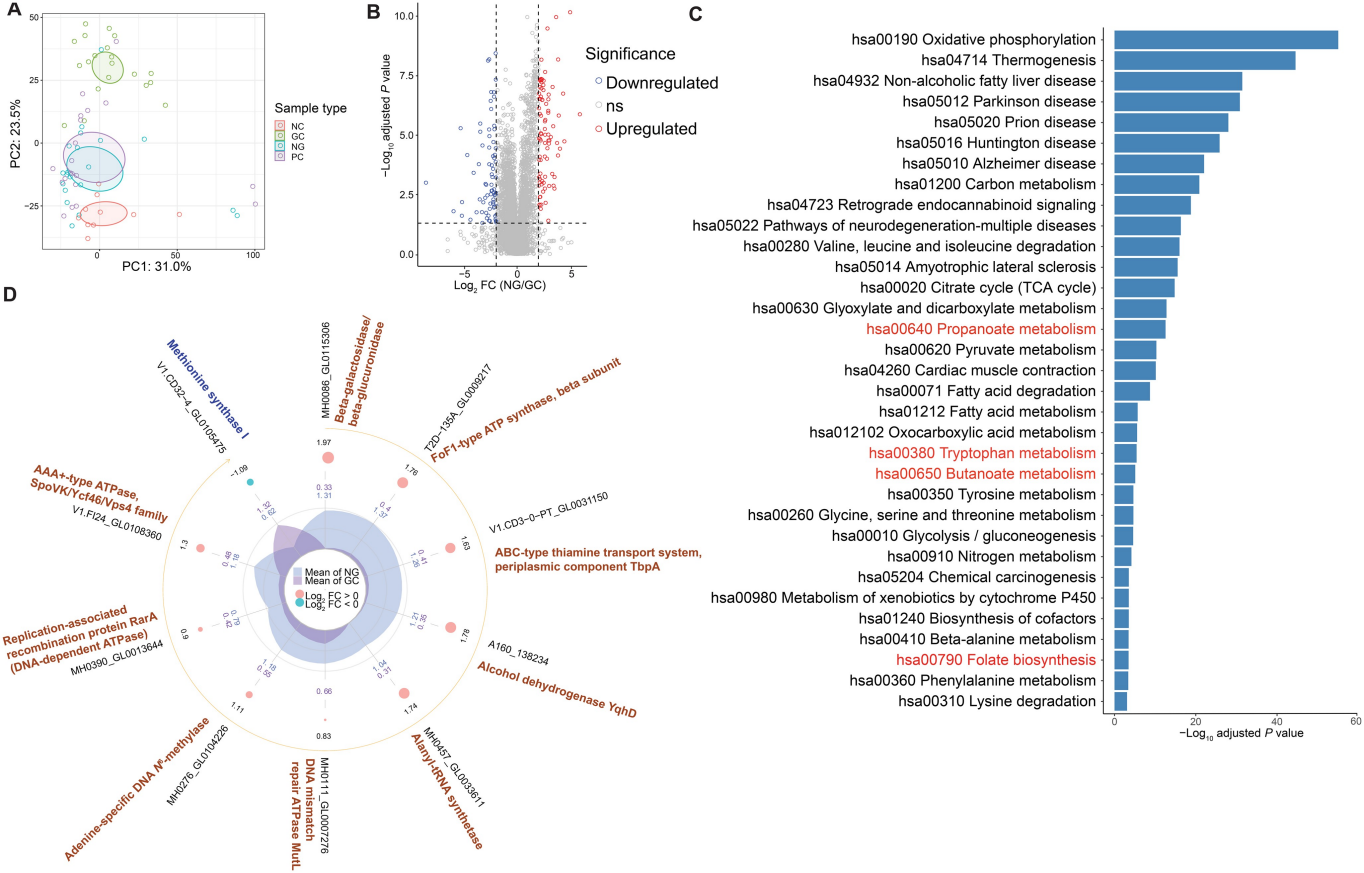
Altered gastric proteomes among groups **A**. PCA plot showing separation among the GC, PC, NG, and NC groups. **B**. Volcano plot of differentially expressed proteins in NG *vs.* GC. The x-axis represents log_2_ FC between NG and GC, and the y-axis depicts −log_10_ adjusted *P* value (Benjamini–Hochberg correction) to control FDR. **C**. KEGG pathway enrichment analysis of differentially expressed proteins in NG *vs.* GC, with *P* values adjusted by the Benjamini–Hochberg method. **D**. Radar chart depicting differential protein expression between GC and NG. The central density plot shows the distribution of mean expression values for GC (light purple) and NG (light blue) groups. Individual features are plotted radially and color-coded by expression change: red dot indicates higher expression in NG (log_2_ FC > 0), blue indicates higher expression in GC (log_2_ FC < 0).

We applied unbiased proteomics analysis to elucidate the potential mechanisms by which intratumoral bacteria influence GC. PCA revealed distinct proteomic clustering between GC and non-tumor tissues (NC, NG, and PC) (**Figure 4**A). Expression of 1086 proteins was significantly downregulated, while 761 proteins were upregulated in NG compared to GC (Figure 4B, |log_2_ FC| > 1.5, *P* < 0.05). Detailed differential expression among groups (NC, NG, PC, and GC) is shown in Figure S6. Briefly, we identified 39 proteins related to host immunity, particularly in the tryptophan metabolic pathway, which were enriched in tumor tissues. In parallel, proteins reported in previous studies [25] to act as potential inhibitors of immune drugs, including GSR, ADA, BCR, ACAT1, POLD1, AKR1C3, and HDAC10, were identified as differentially expressed proteins in our comparative analyses. Their established interactions with drug targets add to the evidence for their potential therapeutic relevance in GC treatment. In addition, 28 proteins related to tryptophan metabolism (ACAT1, HADH, DLD, DLST, MAOA, AFMID, HADHA, MAOB, HAAO, GCDH, ALDH7A1, ECHS1, ALDH3A2, KYAT3, ME3, PDK1, MPC1, GPT, MPC2, PDHX, GSTZ1, ME2, PDK3, ME1, VDAC1, OXSM, MECR, and HSD17B8) were significantly upregulated in the NG group. Notably, expression of proteins that are involved in amino acid and fatty acid synthesis (such as S100B) was also significantly upregulated in the NG group. In contrast, the remaining 25 proteins related to the nod-like receptor signaling pathway (PYCARD, RBCK1, PLCB3, CASP8, IRF9, IFI16, TP53BP1, NAMPT, IRF3, STING1, HSP90AB1, TRPV2, ANTXR1, GBP1, OAS2, CYBB, STAT2, IL18, BCL2L1, CTSB, OAS3, CYBA, STAT1, RIPK2, and CAMP) exhibited increased expression in the GC group (Table S2). Based on proteins upregulated in the NG group, we confirmed that nine proteins are involved in key metabolic pathways, including the ADP, NAD, and Enoyl-CoA hydratase pathways (Table S3). Furthermore, using the Drug-Gene Interaction Database (DGIdb, https://dgidb.org) to analyze drug interactions of target proteins identified in this study, we confirmed SLC25A4, NDUFB8, NDUFA9, SIRT2, and NDUFS2 as potential targets of oncogene inhibitors or their receptors (anti-cancer drugs) (Table S4). Additionally, pathway enrichment analysis showed significant differences (adjusted *P* < 0.05) in multiple pathways between GC and non-tumor tissues (Figure 4C). Mapping the differentially expressed proteins onto the tryptophan metabolism network (https://www.kegg.jp/pathway/map00380) revealed differential expression of metabolites such as IAA, 4,6-dihydroxyquinoline, and kynurenine between GC and non-tumor tissues (Figure S7). These findings suggest that differences in the bacterial community may affect tryptophan metabolism in the host immune system, resulting in changes in relevant metabolites that may be related to tumorigenesis.

We then conducted a meta-proteomic analysis to compare gastric bacterial protein expression between GC and NG tissues. Ten differentially expressed proteins were identified (Figure 4D, |log_2_ FC| > 1.5, *P* < 0.05). The radial visualization (Figure 4D) of these results illustrates the overall expression profiles, with the central density distributions showing distinct mean expression patterns for the NG (light blue) and GC (light purple) groups. Individual proteins are displayed radially and color-coded by differential expression direction: red points (log_2_ FC > 0) indicate proteins upregulated in NG, while blue points (log_2_ FC < 0) represent those downregulated in NG. Among these, methionine synthase I (log_2_ FC < 0) was significantly enriched in the GC group, whereas other proteins, such as alcohol dehydrogenase YqhD, showed higher expression in the NG group. Species prediction and quantitative analysis were performed using the identified peptide sequences. Differentially expressed bacteria (LDA > 2) identified through LEfSe analysis were consistent with those obtained using previous meta-analysis (Figure S8).

### Integrated multi-omics analysis identifies microbiome, proteins, and metabolites involved in gastric carcinogenesis

We performed a comprehensive multi-omics analysis by integrating data on the identified key bacterial taxa, proteins, and metabolites from gastric mucosal tissues derived from amplicon sequencing, as well as metabolomic and proteomic profiling. The integrative multi-omics correlation analysis revealed that 11 pathways were enriched differently between GC and NG. These included taxadiene biosynthesis (engineered), pyruvate fermentation to propanoate I, thiazole biosynthesis II (*Bacillus*), superpathway of thiamin diphosphate biosynthesis II, formaldehyde assimilation II [ribulose monophosphate (RuMP) cycle], superpathway of heme biosynthesis from uroporphyrinogen-III, superpathway of pyrimidine deoxyribonucleoside salvage, superpathway of glucose and xylose degradation, pyrimidine deoxyribonucleosides salvage, tricarboxylic acid (TCA) cycle VIII (*Helicobacter*), and superpathway of menaquinol-8 biosynthesis II. Furthermore, taxadiene biosynthesis (engineered), pyruvate fermentation to propanoate I, thiazole biosynthesis II (*Bacillus*), superpathway of glucose and xylose degradation, and superpathway of menaquinol-8 biosynthesis II were highly enriched in NG compared to GC. Twenty pathways were significantly related to Hp infection and differentially enriched between non-tumor and GC tissues (Spearman correlations, *P* < 0.05). Among these, several pathways, including *L*-histidine biosynthesis, pyruvate fermentation to acetate and lactate II, toluene degradation I (aerobic) (via *o*-cresol), toluene degradation II (aerobic) (via 4-methylcatechol), 5-aminoimidazole ribonucleotide biosynthesis I,5-aminoimidazole ribonucleotide biosynthesis II, CMP-pseudaminate biosynthesis, superpathway of 5-aminoimidazole ribonucleotide biosynthesis, 1,4-dihydroxy-6-naphthoate biosynthesis II, superpathway of demethylmenaquinol-6 biosynthesis II, ADP-*L*-glycero-beta-*D*-manno-heptose biosynthesis, and peptidoglycan maturation (meso-diaminopimelate containing), were upregulated in normal tissues.

**Figure 5:**
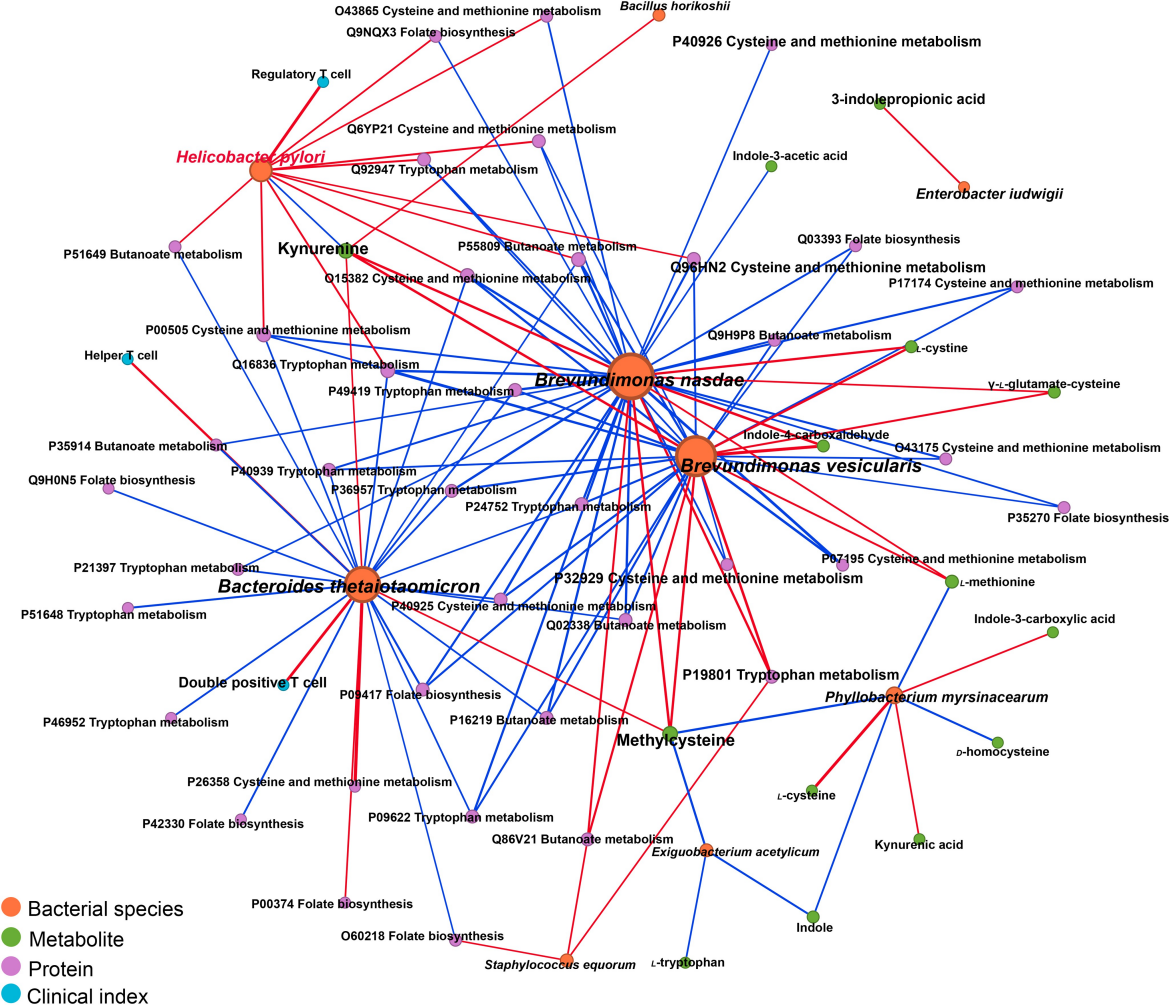
Correlation network of multi-omics and clinical features in GC Network analysis of bacterial species, metabolites, proteins, and clinical indices. Spearman correlations were calculated between each feature, and only those yielding *P* < 0.05 were retained. Correlations are depicted by lines, with positive and negative correlations in red and blue, respectively. Metabolites, bacteria, proteins, and clinical indices are indicated by nodes in green, orange, purple, and blue, respectively.

In addition, we found that the abundance of several bacterial taxa enriched in tumor tissues, such as *B. nasdae*, *B. vesicularis*, and *B. thetaiotaomicron*, was negatively correlated with levels of proteins involved in tryptophan metabolism (P40939, P09622, P21397, P46952, and P51648) and folate metabolism (P42330, P00374, Q9H0N5, P09417, and Q03393). However, kynurenine levels showed a strong positive correlation with these bacterial populations (Figure 5). These taxa were positively correlated with methionine-related proteins and metabolites (*L*-methionine, methylcysteine, *L*-cystine, gamma-glutamate-cysteine, and P26358). Hp was significantly enriched in non-tumor tissues based on meta-analysis and displayed positive correlations with the activation of multiple pathways involved in tryptophan, arginine, folate, and butyrate metabolism, while Hp abundance was negatively correlated with the kynurenine pathway. In parallel, we observed a positive association between Tregs and Hp abundance (Spearman correlations, *P* < 0.05), suggesting that Hp colonization may promote Treg expansion to modulate local immune homeostasis.

## Discussion

Although several studies have reported different states in the bacterial community between GC and non-tumor tissues [24,34], investigations of intragastric bacteria within the same GC individuals with shared genetic backgrounds, dietary habits, and oral/colon bacteria remain limited. The composition of intragastric microbiota can be influenced by dietary factors, as well as cancer-related metabolic processes and drug resistance. However, the precise mechanisms remain under investigation. We performed multi-omics analyses, including full-length 16S rRNA gene sequencing, metabolomics, and proteomics, on matched GC, PC, and NG samples from 20 GC patients and 10 NCs, providing a preliminary framework for understanding how intragastric bacteria may modulate host immune response during GC carcinogenesis. Consistent across both our GC cohort and six external datasets, GC tumor tissues exhibited significantly higher abundance and diversity than NG and PC tissues. At both the genus and species levels, the bacterial communities differ among GC, PC, and NG. Although the core bacterial taxa were detected in all GC tissues, their relative abundances varied, leading to distinct microenvironments even within the same patient.

It has been reported that the relative abundance of Hp is lower in tumors than in non-tumor tissues owing to the decreased acidity of the gastric microenvironment [13,25]. In contrast, our study showed that Hp was more abundant in PC and NG than in GC, particularly in the PC-associated bacterial community. In accordance with a prior investigation, Hp was found to be extremely low in abundance in GC patients compared with non-GC individuals [23]. The decline in Hp population from normal to tumor tissues suggests that its colonization is highly dependent on the stomach microenvironment during gastric carcinogenesis [35,36]. In a healthy stomach, the acidic environment constrains bacterial diversity. Hp infection alters the stomach environment, leading to changes in gastric acidity and inflammation, thereby shaping the intragastric microbiota. This disruption may contribute to the progression of gastric diseases, including cancer, by promoting a microbiome that supports inflammation and carcinogenesis. Our study also reveals intricate interactions among the gastric microbiota, metabolome, and proteome under different tumor microenvironments, particularly in the context of Hp infection. Key metabolic pathways, such as those involved in taxadiene biosynthesis and pyruvate fermentation, were more enriched in non-tumor tissues, reflecting metabolic changes associated with colonization by various bacterial species. These shifts suggest that Hp may influence the gastric environment and disease progression. In tumor tissues, the abundances of bacteria such as *Brevundimonas* and *B. thetaiotaomicron* were negatively correlated with tryptophan- and folate-metabolism proteins but positively correlated with kynurenine- and methionine-related metabolites, suggesting that specific microbial populations may contribute to metabolic alterations linked to GC development. Additionally, the abundance of Hp showed positive correlations with proteins involved in tryptophan, arginine, folate, and butyrate metabolism, as well as host Tregs, highlighting its role in modulating immune and metabolic pathways. However, due to the small sample size, it is challenging to determine the precise roles of individual gastric bacteria in tumorigenesis, and the controversial distribution of probiotics in the stomach and gut still needs to be further investigated. More samples with Hp infection status available for all samples would help to explain the interaction between Hp infection and intragastric microbiota.

Multiple pathways, including cell cycle, protein kinase signaling, immune responses, and chemokine activity, are involved in chronic gastric inflammation that induces GC [25,28]. Based on correlation analysis, we found that the signaling pathways of stem cells regulating pluripotency in GC were upregulated, while they were largely inactive in non-tumor samples. The pathway of glycan degradation was more prominent in PC, indicating energy-dynamic shifts during gastric carcinogenesis [37,38]. In addition, the pathway of epithelial cell signaling with Hp infection peaked in PC, and was similar in GC and NC in the present work. Previous studies have reported that the pathways related to antioxidation and tumor growth were highly expressed in GC tumors [39,40]. Metabolites from intragastric bacteria have been reported to act differently in cancerous and non-tumor tissues, even within the same GC patient, likely due to variations in diet and health conditions among individuals [25,28]. In our cohort, for GC and non-tumor tissues from the same patient, several metabolites involved in amino acid synthesis and metabolism were more abundant in GC than in PC or NG, aligning with host immune pathways implicated in gastric carcinogenesis, such as the tryptophan pathway [41]. Notably, metabolites related to the tryptophan pathway were upregulated in normal tissues, including indoleamine 2,3-dioxygenase (IDO), which regulates host immunity by catalyzing tryptophan to kynurenine, thereby exerting immunomodulatory effects [42]. Immune system dysfunction, such as T lymphocytes depletion observed in GC patients, can act in concert with changes in the tumor microenvironment. Inflammation may enhance tumorigenesis by inhibiting anti-tumor immunity and remodeling the microenvironment to favor tumor cell growth [43].

We found that *Bifidobacterium* was enriched in normal tissues, consistent with its reported anticancer benefits [44,45], enhancement of PD-L1 and CTLA-4 blockade, dendritic cell activation, and CD8^+^ T-cell tumor targeting [46]. In our study, *Prevotella* showed higher abundance in GC tissues, which influences the expression of IL-2 and IFN-γ, regulates the metabolism of glycerophospholipids, and reduces the immunotherapeutic response to PD-1 inhibitors [47,48]. By correlating the relative abundance of gastric bacteria and differentially expressed proteins, we found that the status of Hp, *B. nasdae*, *B. thetaiotaomicron*, and *B. vesicularis* in different tumor microenvironments was linked to the activity of the kynurenine and serotonin pathways. Aside from gut and oral bacteria, the gastric microbiota plays an essential role in regulating host immunity via multiple routes, including the production of short-chain fatty acids (SCFAs) and modulation of the tryptophan metabolic pathway [49]. An imbalance in tryptophan metabolism could enhance the development of Hp-associated GC by interfering with host immune responses under oxidative stress. The Hp infection can promote oxidative stress, leading to DNA damage and inflammation, and creating an environment conducive to GC development. Moreover, Hp-induced oxidative stress promotes the conversion of tryptophan to kynurenine, thereby suppressing the immune response by increasing the generation of Tregs [50–52]. In our investigation, the abundance of *B. nasdae* was negatively correlated with IAA, whereas the abundance of *Phyllobacterium myrsinacearum* was positively correlated with indole-3-carboxylic acid. These metabolic and protein alterations may perturb multiple immune pathways. Depending on their group status (NG, GC, or PC), certain bacterial species may serve as biomarkers for early diagnosis or as targets for microbial treatment of GC. However, further mechanistic studies are needed to elucidate the underlying processes governing these associations and to explore potential therapeutic targets for these diseases.

## Conclusion

Understanding the relationship between the immune system and gastric microbial homeostasis is essential for early diagnosis and the development of effective therapies for GC. Our findings indicate that the interplay among the gastric mucosal microbiota, metabolites, and proteins during GC progression forms a complex, interconnected network. While the study provides valuable insights into interactions between the gastric microbiota, metabolome, and proteome, several limitations should be acknowledged. The data validation was primarily based on 16S rRNA sequencing, which characterizes microbial composition but lacks direct metabolomic and proteomic evidence. Consequently, although correlations between microbial populations and metabolic pathways were identified, further validations are needed *in vivo* and *in vitro*. Future studies should incorporate these additional layers of validation to substantiate the functional implications of the observed microbial and metabolic interactions.

## Materials and methods

### Cohort recruitment and sample collection

Twenty GC patients who underwent gastrectomy between January 2018 and August 2019 at the Fifth Affiliated Hospital, School of Medicine, Zhengzhou University, China were enrolled in this study. NG samples were collected 5 cm from the matched GC tissues in the gastric mucosa. Patients were diagnosed by postoperative pathological examination, and clinical staging was determined according to the 8th edition of the American Joint Committee on Cancer (AJCC) cancer staging manual for GC using the Tumor, Node, Metastasis (TNM) staging system. Patients receiving antibiotics within three months, drug or alcohol addiction, or a history of abdominal cancer were excluded from this study. Ten obese subjects free of GC, gastric precancerous lesions, or other gastrointestinal disorders were enrolled as NCs, given their suitability for convenient sample collection. Their gastric mucosal histology was confirmed normal, and no medications affecting gastric immunity or metabolism were administered. These clinical characteristics ensured that the NC group provided valid non-tumor tissue references for comparing with GC tissues. Demographic and clinicopathological characteristics of GC patients are summarized in Table S1.

### Public data acquisition and meta-analysis

A comprehensive literature search was performed in PubMed to identify studies exploring the characteristics and role of the mucosal microbiota in GC. The search query employed a combination of Medical Subject Headings (MeSH) terms and free-text keywords to maximize sensitivity: [“mucosal microbiota” (Title/Abstract) OR “gastric microbiome” (Title/Abstract) OR “stomach microbiota” (Title/Abstract) OR “Microbiota” (MeSH)] AND [“Stomach Neoplasms” (MeSH) OR “gastric cancer” (Title/Abstract) OR “gastric carcinoma” (Title/Abstract) OR “GC” (Title/Abstract)]. Raw FASTQ files from six external datasets were downloaded from the European Nucleotide Archive (ENA), with accession Nos. PRJNA310127, PRJNA428883, NMDC10017675, PRJEB21497, PRJNA239281, and PRJNA413125. Data for chronic gastritis and gastric carcinoma were collected from PRJNA239281 and PRJNA41312, respectively. Data for paired tumor and non-tumor mucosal tissues of GC patients were collected from NMDC10017675, PRJNA428883, and PRJNA310127. All raw sequencing data were reprocessed using the Divisive Amplicon Denoising Algorithm 2 (DADA2) plugin within the Quantitative Insights Into Microbial Ecology 2 (QIIME2) pipeline for taxonomic profiling. Meta-analysis was conducted using the code available at https://github.com/zellerlab/crc_meta [53].

### Full-length 16S rRNA gene amplicon sequencing and data analysis

Total bacterial genomic DNA was extracted using sodium dodecyl sulfate, and the concentration and purity of the extracted DNA samples were measured with NanoDrop ND-2000 spectrophotometer (Catalog No. ND-2000, Thermo Fisher Scientiﬁc, Waltham, MA) and agarose gel electrophoresis. Full-length bacterial 16S rRNA gene sequences were amplified using the forward primer 27F (5′-AGRGTTYGATYMTGGCTCAG-3′) and reverse primer 1492R (5′-RGYTACCTTGTTACGACTT-3′) with sample-specific 16-bp barcodes. PCR mixture consisted of 5 μl of KAPA HiFi buffer (5×), 0.75 μl of KAPA HiFi hot start DNA polymerase (1 U/μl), 0.75 μl (10 mM) of dNTPs, 0.75 μl of primer (10 μM; forward or reverse), 2 μl of DNA template, and 15 μl of ddH_2_O. The PCR conditions were as follows: initial denaturation at 95°C for 5 s, followed by 25 cycles consisting of denaturation at 95°C for 30 s, annealing at 57°C for 30 s, and extension at 72°C for 60 s, and a final extension at 72°C for 5 min. PCR amplicons were purified using Agencourt AMPure Beads (Catalog No. A63880, Beckman Coulter, Indianapolis, IN) and quantified using the PicoGreen dsDNA assay kit (Catalog No. P7589, Invitrogen, Carlsbad, CA). The amplicons were then pooled in equal amounts to generate a library using the SMRTbell Template Prep Kit 1.0-SPv3 (Catalog No. 100-222-300, Pacific Biosciences, Menlo Park, CA). The library was evaluated using a Qubit 3.0 Fluorometer (Catalog No. Q33216, Life Technologies, Carlsbad, CA) for the quality and sequenced on the PacBio platform with the DNA/Polymerase Binding Kit 2.0 (Catalog No. 101-789-500, Pacific Biosciences) at Wuhan Frasergen Bioinformatics Co., Ltd. (Wuhan, China).

Since the raw data from PacBio SMRT sequencing contained a certain proportion of random errors, we applied self-alignment to obtain high-quality circular consensus sequences (CCS), followed by removing chimeric sequences to yield clean data. The operational taxonomic units (OTUs) were clustered at the species level, and the OTU abundance and diversity were analyzed.

The alpha diversity analysis was performed to evaluate microbial richness within samples, and beta diversity among groups was assessed by PCoA. We then used MaAsLin2 (*q* < 0.3) and the two-proportions Z-test (*P* < 0.05) to identify differential features among groups. In addition, PICRUSt2 was used to predict metabolic pathways associated with GC.

### Tissue sample preparation and LC-MS conditions for metabolomics analysis

Frozen tissue samples (100 mg) were thawed on ice and then ground at 30 Hz for 3 min. The homogenates were resuspended in 1 ml of 70% methanol, incubated on ice for 5 min, and centrifuged at 12,000 r/min for 10 min at 4°C. The supernatants were transferred to an Eppendorf tube, stored at −20°C overnight, and then centrifuged again at 12,000 r/min at 4°C for 3 min. Finally, the supernatant was injected into the LC-MS system.

A Waters ACQUITY UPLC HSS T3 C18 column (1.8 μm, 2.1 mm × 100 mm; Catalog No. 186003538, Genetech Scientific, Arcade, NY) was used for chromatographic separation. The column temperature was maintained at 40°C, and the flow rate was 0.4 ml/min. Gradient elution was performed using mobile phase A (0.1% formic acid in water) and mobile phase B (0.1% formic acid in acetonitrile). The solvent gradient was set as follows: 95:5 (A/B, v/v) at 0 min, 10:90 (A/B, v/v) at 10.0 min, 10:90 (A/B, v/v) at 11.0 min, 95:5 (A/B, v/v) at 11.1 min, and 95:5 (A/B, v/v) at 14.0 min. The injection volume was 5 μl.

A Triple TOF mass spectrometer (Triple TOF 6600, AB SCIEX Marlborough, MA) was used for full-scan mass detection. A precursor ion with an intensity greater than 100 was selected for subsequent tandem mass spectrometry (MS/MS) fragmentation with a collision energy (CE) of 30 eV. An ESI source was used for mass analysis. The MS operation parameters were set as follows: ion source gas at 50 pound-force per square inch (psi); curtain gas at 25 psi; source temperature at 500°C; ion spray voltage floating (ISVF) in positive and negative modes at 5500 V and −4500 V, respectively.

Linear ion trap (LIT) and triple quadrupole (QQQ) scans were acquired using a triple quadrupole-linear ion trap mass spectrometer (QTRAP LC-MS/MS System) equipped with an ESI Turbo Ion-Spray interface. Both positive and negative ion modes were conducted in the ESI source for mass analysis, controlled by the Analyst 1.6.3 software (Sciex). The ESI source operation parameters were set as follows: source temperature 500°C; ion spray voltage (IS) for positive and negative mode at 5500 V and −4500 V, respectively; ion source gas I (GSI), gas II (GSII), and curtain gas (CUR) were set at 50, 50, and 25.0 psi, respectively. Instrument tuning and mass calibration were performed with 10 and 100 μM polypropylene glycol solutions in QQQ and LIT modes.

Quality control (QC) samples were prepared by aliquoting 10 µl from each specimen for quality assessment. Commencing with the doNGstream analysis of the metabolomic data, two criteria were employed to filter out noisy features: the “80% rule”, which requires a feature to be present in at least 80% of the samples within any given group, and the “30% Rule of Quality”, *i.e.*, a feature’s relative standard deviation (RSD) in QC samples must be less than 30%. A PCA plot was conducted to visualize the feature distribution and assess the clustering of QC samples. A tight aggregation of QC samples on the PCA plot indicated the methodological and data consistency. Further, differential metabolites were identified using a variable selection procedure within sPLS-DA, retaining those with a significance threshold of *P* < 0.05.

### Protein extraction, tryptic digestion, and LC-MS conditions for proteomics analysis

Tissues were ground with liquid nitrogen, and the homogenates were resuspended in lysis buffer [1% Triton X-100, 1% protease inhibitor, 1% phosphatase inhibitors, 50 μM PR-619, 3 μM Trichostatin A (TSA), and 50 mM nicotinamide (NAM)]. The lysates were centrifuged at 12,000 r/min for 10 min at 4°C. The supernatants were transferred to a new Eppendorf tube, and the protein concentration was measured using the BCA method.

Proteins were replenished with 20% trichloroacetic acid. After 2 h incubation (4°C), samples were centrifuged at 4500 r/min for 5 min at 4°C. The pellets were washed three times with ice-cold acetone, air dried, and resuspended in 200 mM triethylammonium bicarbonate. Trypsin digestion was performed overnight at an enzyme-to-protein mass ratio of 1:50. Samples were supplemented with dithiothreitol to a final concentration of 5 mM and maintained at 56°C for 30 min, then supplemented with 11 mM IAA and incubated at room temperature in the dark for 15 min.

Digested peptides were analyzed using NanoElute 2, a high-performance nanoflow LC system. The peptide samples were separated using gradient elution, with mobile phase A (0.1% formic acid in water/acetonitrile, 98:2 v/v) and mobile phase B (0.1% formic acid in acetonitrile). The solvent gradient was: 0–70 min, 6%–24% of B; 70–84 min, 24%–35% of B; 84–87 min, 35%–80% of B; 87–90 min, 80% of B. The injection volume was 5 μl. The flow rate was 450 nl/min.

LC-MS-based proteomics analysis was conducted using timsTOF Pro, which couples trapped ion mobility spectrometry (TIMS) with high-resolution TOF MS. The ion-source voltage was set to 1.75 kV. The parallel accumulation–serial fragmentation (PASEF) mode was used to acquire the data. The scan range was *m/z* 100–1700. The dynamic exclusion time of the previously obtained precursor ions was 30 s, and the cycle time was 1 s.

Species prediction and quantitative analysis were performed using the identified peptide sequences. The Unipept (v2.2.1) tool was utilized to predict species information for each peptide segment using the Lowest Common Ancestor (LCA) algorithm. The relative abundance of each species in the sample was calculated based on the proportion of the intensity of the peptides assigned to that species over the total intensity of all the identified peptides. Subsequently, the predicted species composition and relative abundance information were used for species composition visualization, species diversity analysis, and differential species analysis.

### Statistical analysis

Statistical analyses and figure generation were performed using R software (v3.6.1, http://www.r-project.org/). The AUROC with permutation-based confidence intervals was calculated using the “pROC” package in R as a non-parametric effect size measure. The Mann–Whitney U test was used to assess group differences. Statistical significance was defined as *P* < 0.05, with *P* values adjusted for multiple testing via the Benjamini–Hochberg method, denoted as *q* values. Microbiome–metabolome correlation analysis was performed using Spearman’s rank correlation method.

ANOVA was performed to quantify the contribution of potential confounding factors relative to tumor status for individual microbial species. The variance explained by tumor status and the confounding factor was compared with the total variance in the abundance of a given microbial species, akin to a linear model. To accommodate the non-Gaussian distribution of gastric microbiome abundance data, microbial diversity and dynamics were analyzed using MaAsLin2 with a *q* value cutoff of 0.3 to ensure robust statistical relevance. A two-proportions Z-test was also applied with a significance threshold of *P* < 0.05. Subsequently, sPLS-DA was conducted to identify differential metabolites, with a significance threshold of *P* < 0.05.

## Supplementary Material

qzaf132_Supplementary_Data

## Data Availability

The 16S rRNA data generated in this study have been deposited in the BioProject database at the NGDC, CNCB (BioProject: PRJCA042633), which is publicly accessible at https://ngdc.cncb.ac.cn/bioproject/browse/PRJCA042633. The proteomic and metabolomic datasets are provided in Tables S5 and S6, respectively.

## Code availability

The code has been submitted to BioCode at the National Genomics Data Center (NGDC), China National Center for Bioinformation (CNCB) (BioCode: BT008049), which is publicly accessible at https://ngdc.cncb.ac.cn/biocode/tools/8049.

## CRediT author statement

**Pengyuan Zheng:** Conceptualization, Project administration, Funding acquisition, Supervision. **Xiaochen Yin:** Conceptualization, Project administration, Funding acquisition, Supervision. **Yang Mi:** Writing – original draft, Methodology, Software. **Die Dai:** Writing – original draft, Methodology, Software. **Xia Xue:** Writing – original draft, Methodology, Software. **Xiaojie Li:** Writing – original draft, Methodology, Software. **Haiming Qin:** Methodology, Validation, Writing – review & editing. **Feifei Ren:** Methodology, Validation, Writing – review & editing. **Xin Xie:** Methodology, Validation, Writing – review & editing. **Ihtisham Bukhari:** Methodology, Validation, Writing – review & editing. **Shaogong Zhu:** Methodology, Validation, Writing – review & editing. **Yan Tan:** Methodology, Validation, Writing – review & editing. **Barry J. Marshall:** Supervision, Writing – review & editing. **Alfred Tay:** Supervision, Writing – review & editing. **Youcai Tang:** Supervision, Writing – review & editing. **Haiqing Bai:** Resources, Formal analysis, Validation. **Yong Yu:** Resources, Formal analysis, Validation. **Wanqing Wu:** Resources, Formal analysis, Validation. All authors have read and approved the final manuscript.

## ORCID

0000-0003-2905-4830 (Yang Mi)

0000-0001-5309-8046 (Die Dai)

0000-0002-5783-1281 (Xia Xue)

0009-0002-6509-3984 (Haiming Qin)

0000-0002-8751-5677 (Feifei Ren)

0000-0003-4853-5015 (Barry J. Marshall)

0000-0001-9705-4010 (Alfred Tay)

0000-0002-0728-1840 (Ihtisham Bukhari)

0009-0006-8228-6189 (Xiaojie Li)

0009-0006-5682-2240 (Shaogong Zhu)

0009-0006-6429-9234 (Yong Yu)

0009-0002-4628-6959 (Wanqing Wu)

0000-0001-9656-8861 (Yan Tan)

0009-0004-4334-0635 (Youcai Tang)

0009-0004-2002-3944 (Xin Xie)

0000-0003-0865-1126 (Haiqing Bai)

0009-0007-3781-0604 (Xiaochen Yin)

0000-0003-1647-0253 (Pengyuan Zheng)
