# Supplementary material for: Multi-omics Analysis Reveals How Intratumoral Bacteria Shape the Immune Microenvironment in Gastric Cancer
Source: Genomics Proteomics Bioinformatics. 2025 Dec 27;23(6):qzaf132. doi: 10.1093/gpbjnl/qzaf132 (PMC13197131; doi:10.1093/gpbjnl/qzaf132)
Supplement: qzaf132_Supplementary_Data [file qzaf132_supplementary_data.zip › Figure S1.pdf]

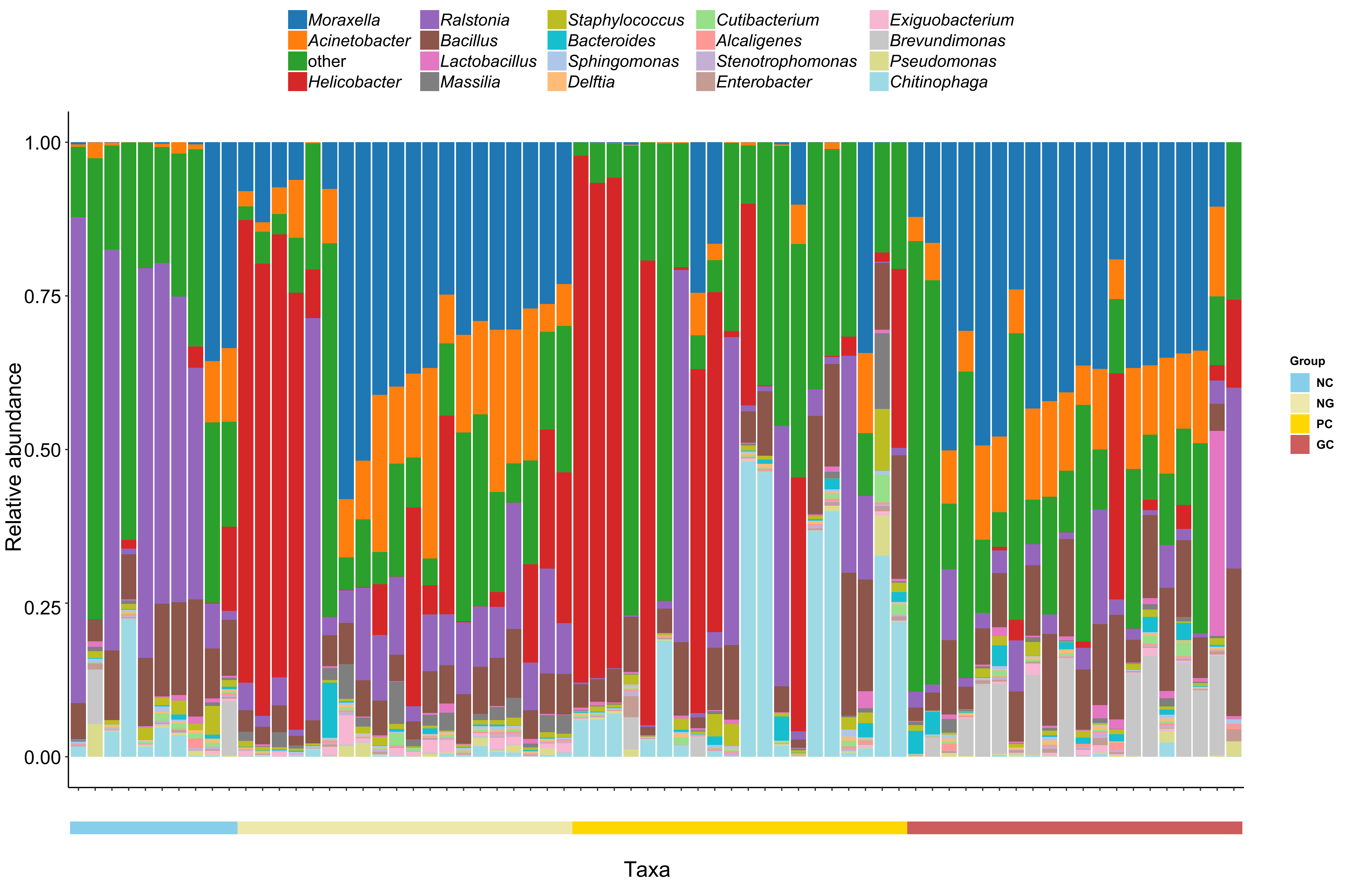

Moraxella
Acinetobacter
other
Helicobacter
Ralstonia
Bacillus
Lactobacillus
Massilia
Staphylococcus
Bacteroides
Sphingomonas
Delftia
Cutibacterium
Alcaligenes
Stenotrophomonas
Enterobacter
Exiguobacterium
Brevundimonas
Pseudomonas
Chitinophaga
Relative abundance
1.00
0.75
0.50
0.25
0.00
Group
NC
NG
PC
GC
Taxa
