# Supplementary material for: Multi-omics Analysis Reveals How Intratumoral Bacteria Shape the Immune Microenvironment in Gastric Cancer
Source: Genomics Proteomics Bioinformatics. 2025 Dec 27;23(6):qzaf132. doi: 10.1093/gpbjnl/qzaf132 (PMC13197131; doi:10.1093/gpbjnl/qzaf132)
Supplement: qzaf132_Supplementary_Data [file qzaf132_supplementary_data.zip › Supplementary material captions.docx]

**Supplementary material**

**Figure S1 Phylogenetic composition of common microbial taxa at the genus level**

Phylogenetic composition was ordered by groups (NC, NG, PC, GC) and by the most abundant taxa. GC, gastric cancer; PC, para-carcinoma; NG, normal gastric mucosa; NC, non-cancer control.

**Figure S2 Heatmap of differentially abundant genus between groups**

Each tile represents the scaled relative abundance of a taxon. Red dots in the right plot denote the bacteria that differ significantly between groups, based on MaAsLin2 analysis (*q* < 0.3) and two-proportions Z-test (*P* < 0.05). The *q* value denotes the adjusted *P* value.

**Figure S3 Diversity and composition of the gastric mucosal microbiota across seven independent datasets**

**A.** Alpha diversity (Shannon index) of the gut microbiota across datasets P1-P7. The *P* values were computed using a two-sided Wilcoxon test. **B.** Principal coordinate analysis based on Bray-Curtis distance, depicting the microbial community composition of samples from the seven included datasets. Samples are color-coded by study and shape-coded by tumor status (solid circles for GC samples, hollow circles for non-tumor samples). The accompanying boxplots display the distribution of samples projected onto the first two principal coordinates, stratified by study and by tumor status, respectively.

**Figure S4**  **AUROC analysis of core significant bacteria in GC and non-tumor Groups**

For a core of highly significant bacteria (meta-analysis FDR = 0.001), AUROC was calculated for GC *versus* non-tumor groups across individual datasets (color-coded diamonds), with 95% confidence intervals indicated by the gray lines.

**Figure S5 Signature bacteria in tumor tissues compared to non-tumor tissues in GC patients**

Gastric bacterial abundance from six independent datasets and the present dataset was determined. Blue box refers to the relative abundance of the bacterial genus in non-tumor tissues, and red denotes tumor tissues.

**Figure S6 Number of differentially expressed proteins identified between groups**

**Figure S7 Mapping of differentially expressed proteins in the tryptophan metabolism pathway**

Differentially expressed proteins in the tryptophan metabolism pathway were mapped onto the metabolic network diagram, with red indicating enrichment in non-tumor tissue and green indicating enrichment in tumor tissue.

**Figure S8 Differentially abundant bacteria (LDA > 2) identified through LEfSe analysis**

**A.** Differentially abundant bacteria between GC and NG; **B.** Differentially abundant bacteria between GC and PC. LDA, linear discriminant analysis; LEfSe, linear discriminant analysis Effect Size.

**Table S1 Clinicopathological characteristics of gastric cancer (GC) patients in this study**

**Table S2 Significantly differentially expressed proteins between normal gastric mucosa (NG) and gastric cancer (GC) groups**

**Table S3 KEGG annotation of differentially expressed proteins between normal gastric mucosa (NG) and gastric cancer (GC) groups**

**Table S4 Drug interactions of target proteins identified in this study using DGIdb**

**Table S5 Data for metabolomics analysis**

**Table S6 Data for proteomics analysis**
