# Supplementary material for: Multi-omics Analysis Reveals How Intratumoral Bacteria Shape the Immune Microenvironment in Gastric Cancer
Source: Genomics Proteomics Bioinformatics. 2025 Dec 27;23(6):qzaf132. doi: 10.1093/gpbjnl/qzaf132 (PMC13197131; doi:10.1093/gpbjnl/qzaf132)
Supplement: qzaf132_Supplementary_Data [file qzaf132_supplementary_data.zip › Table S1.docx]

**Table S1 Clinicopathological characteristics of gastric cancer (GC) patients in this study**

| **Characteristics** | **GC patients** |
| --- | --- |
| **Total number** | 20 |
| Female | 3 |
| Male | 17 |
| **Age (years, mean)** | 64.4+5.98 |
| **Comlications** |  |
| Hypertension | 2 |
| Diabetes mellitus | 1 |
| **Tumor localization** |  |
| Proximal stomach | 4 |
| Antrum | 5 |
| Body/fundus | 12 |
| **Tumor differentiation** |  |
| Mederately-highly differentiated | 2 |
| Mederately differentiated | 8 |
| Moderately-poorly differentiated | 2 |
| Poorly differentiated | 5 |
| Unknown | 3 |
| **Lauren typing** |  |
| Intestinal type | 7 |
| Diffuse type | 4 |
| Mixed type | 2 |
| **Tumor stage** |  |
| I | 4 |
| II | 7 |
| III | 7 |
| Ⅳ | 2 |
| **Total lymphocyte CD45^+^ (tissue-infiltrating lymphocytes)** | 15 ± 0.22 |
| **PD-1+Tc (CD8^+^CD279^+^)** | 2.55 ± 2.07 |
