# Supplementary figures and images for: Multi-omics Analysis Reveals How Intratumoral Bacteria Shape the Immune Microenvironment in Gastric Cancer
Source: Genomics Proteomics Bioinformatics. 2025 Dec 27;23(6):qzaf132. doi: 10.1093/gpbjnl/qzaf132 (PMC13197131; doi:10.1093/gpbjnl/qzaf132)

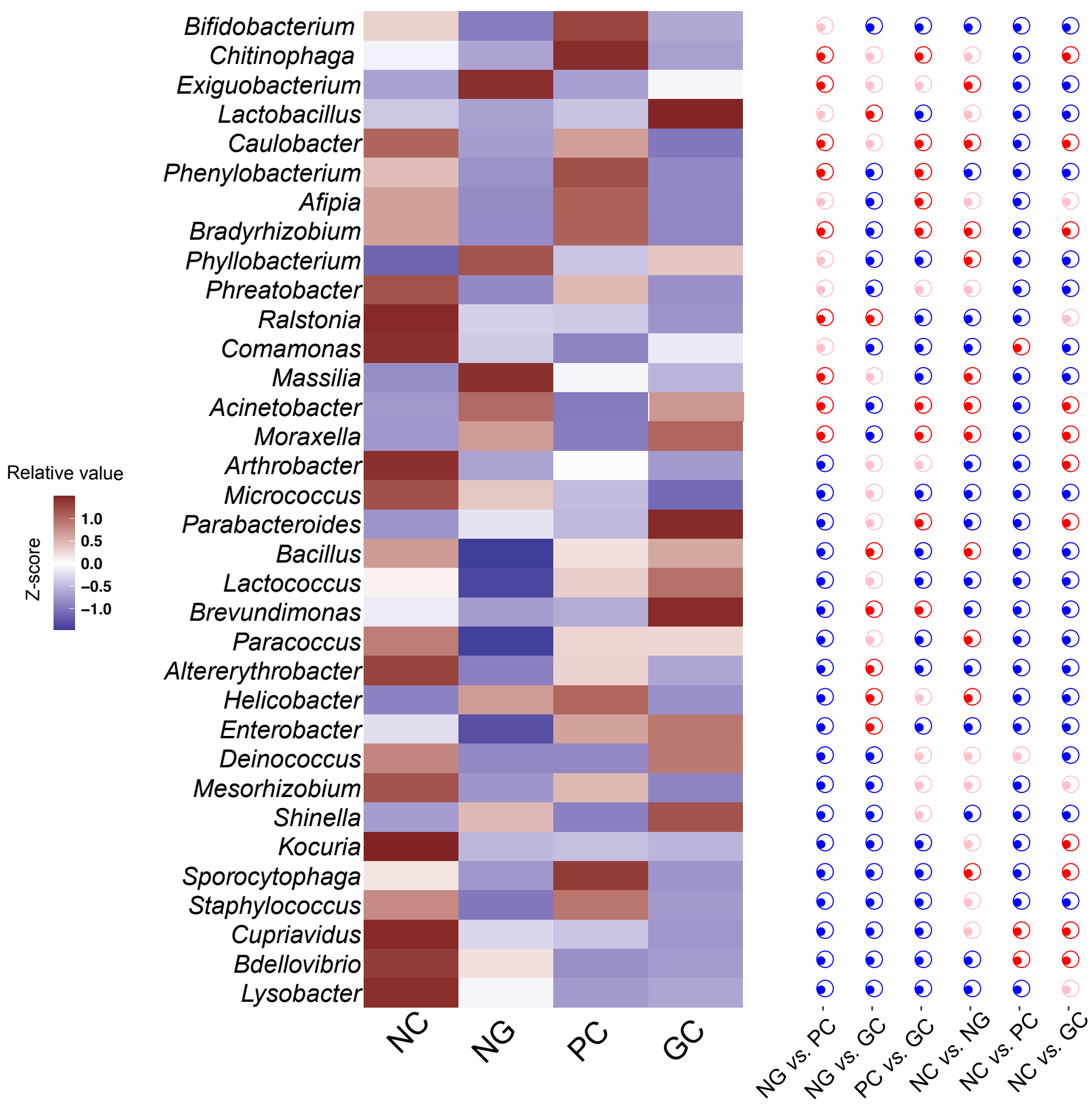

Supplement: qzaf132_Supplementary_Data [file qzaf132_supplementary_data.zip › Figure S2.pdf]

**A**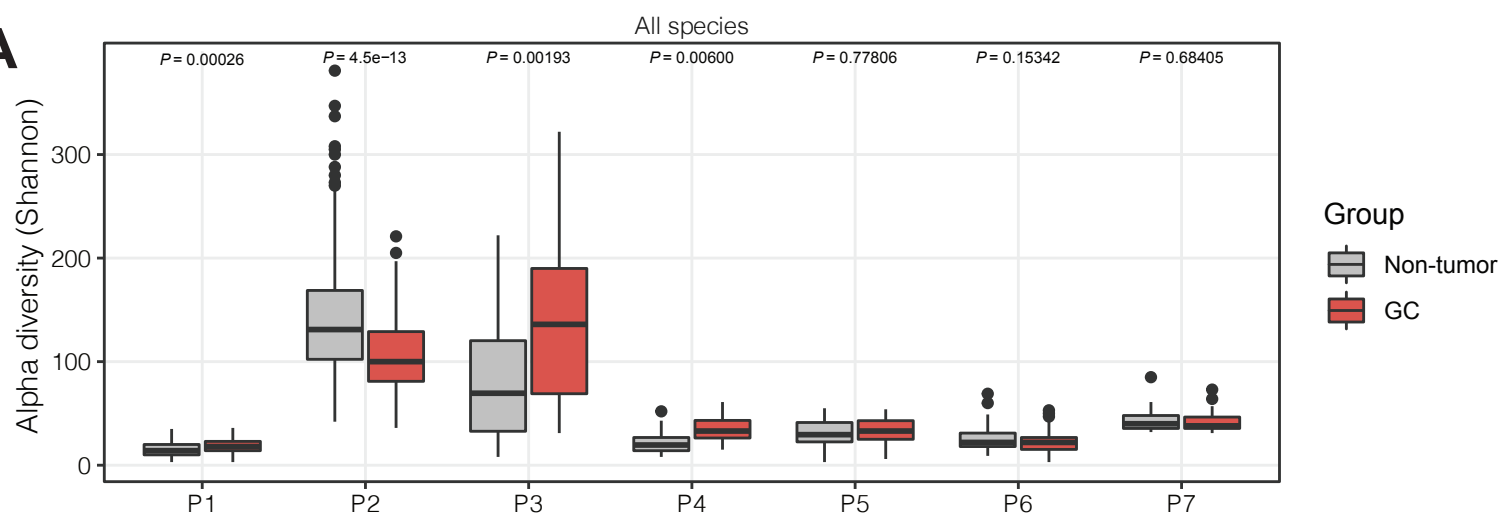**B**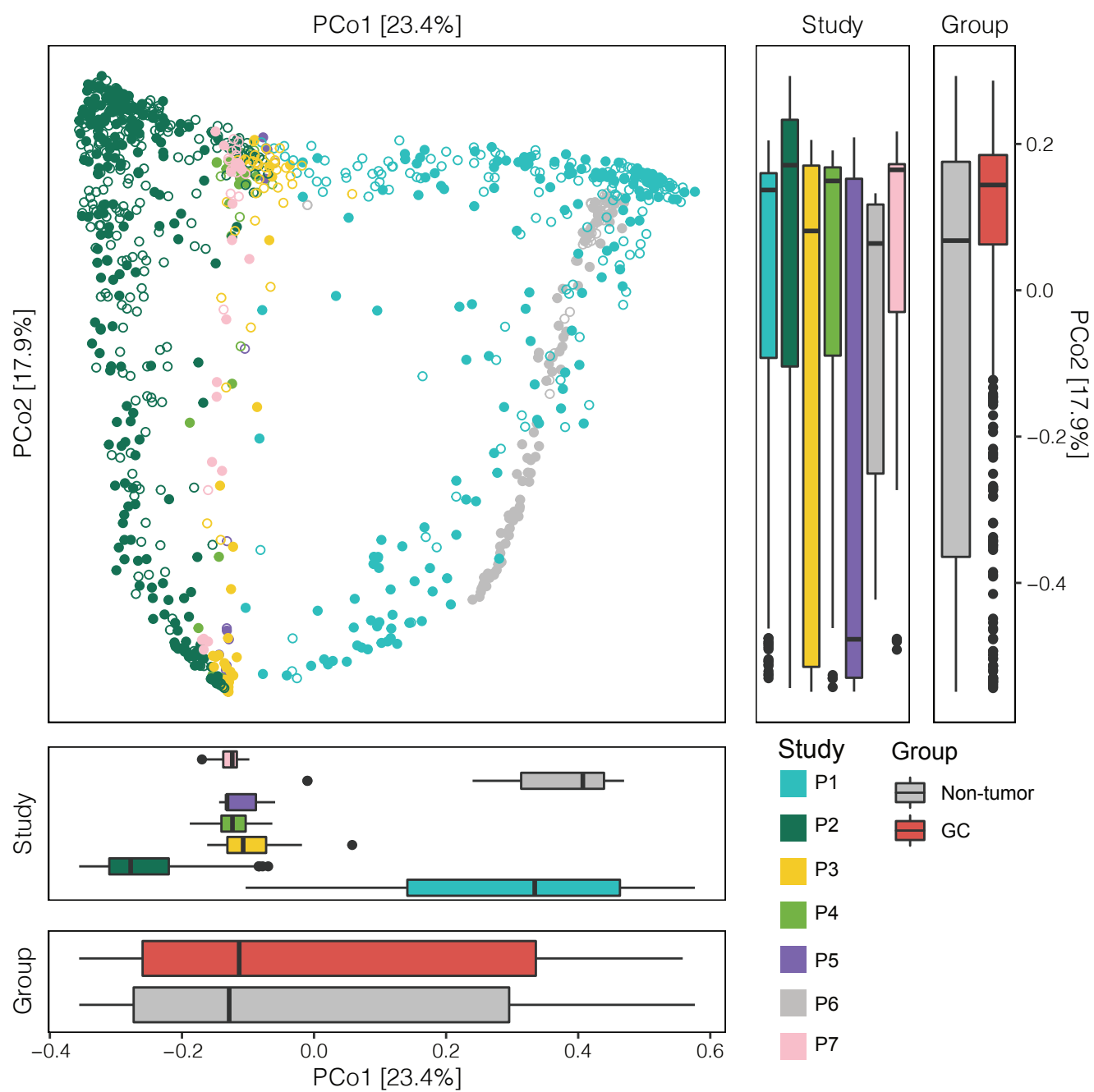

Supplement: qzaf132_Supplementary_Data [file qzaf132_supplementary_data.zip › Figure S3.pdf]

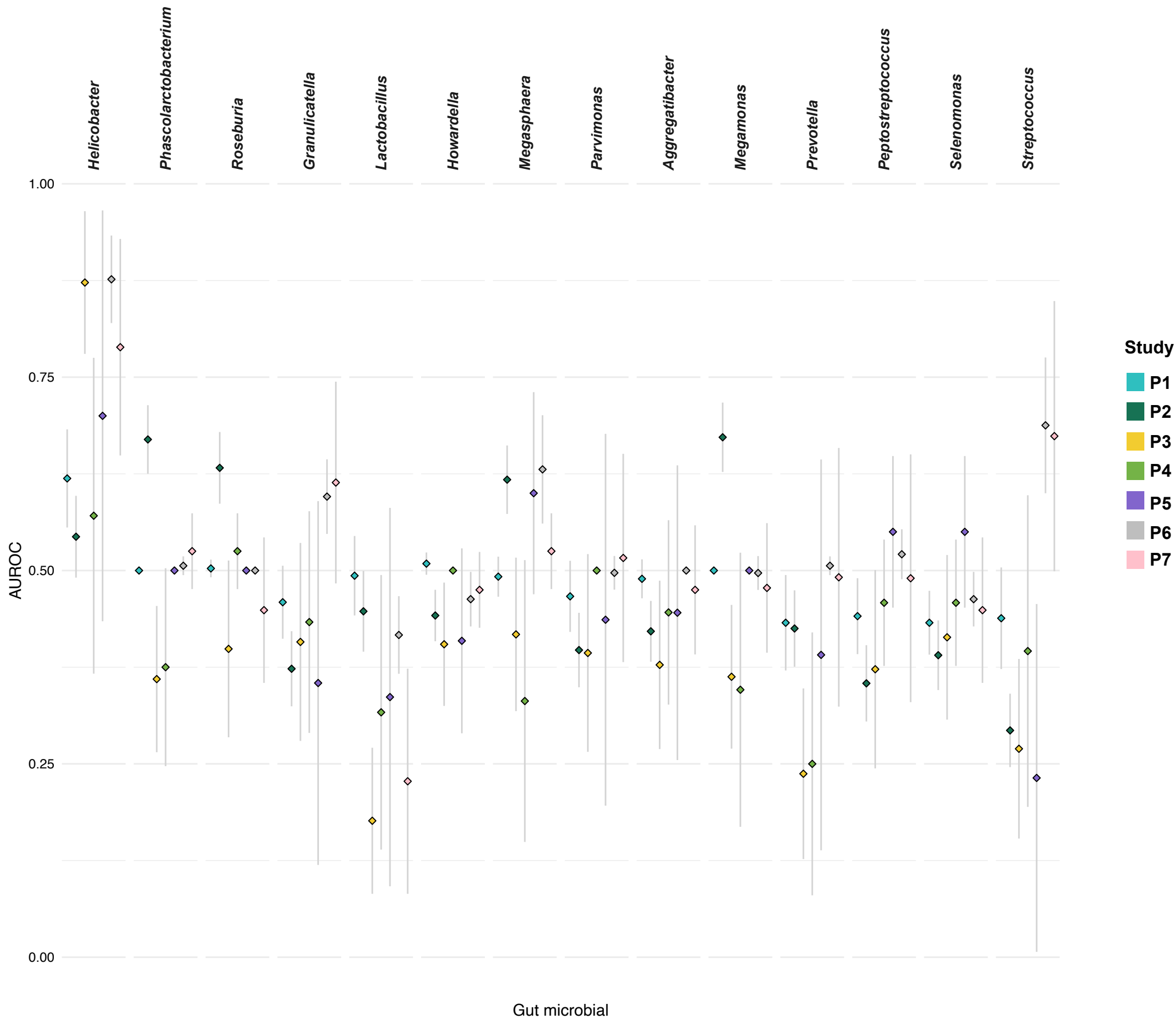

Supplement: qzaf132_Supplementary_Data [file qzaf132_supplementary_data.zip › Figure S4.pdf]

## *Lactobacillus*

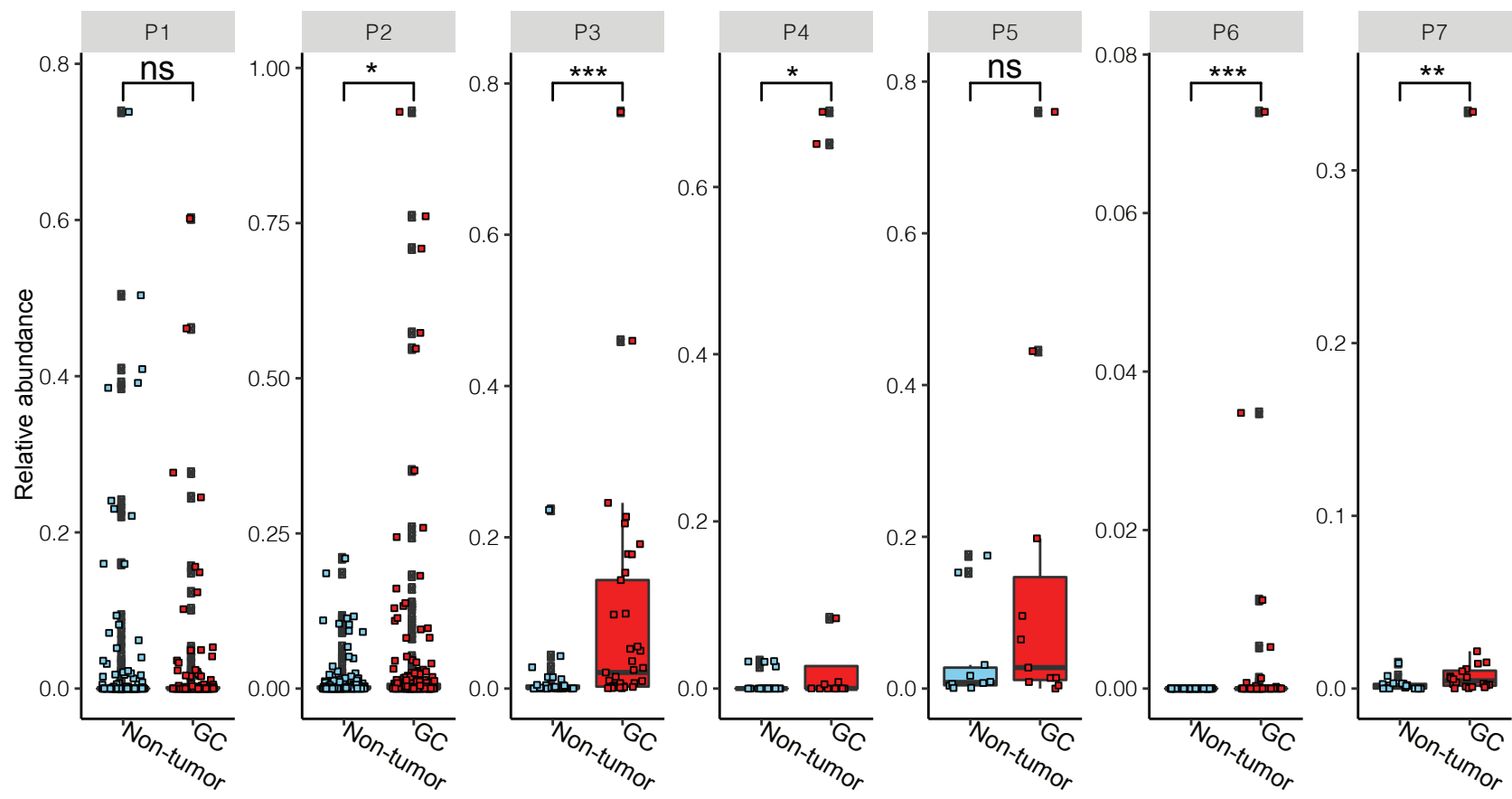

## *Prevotella*

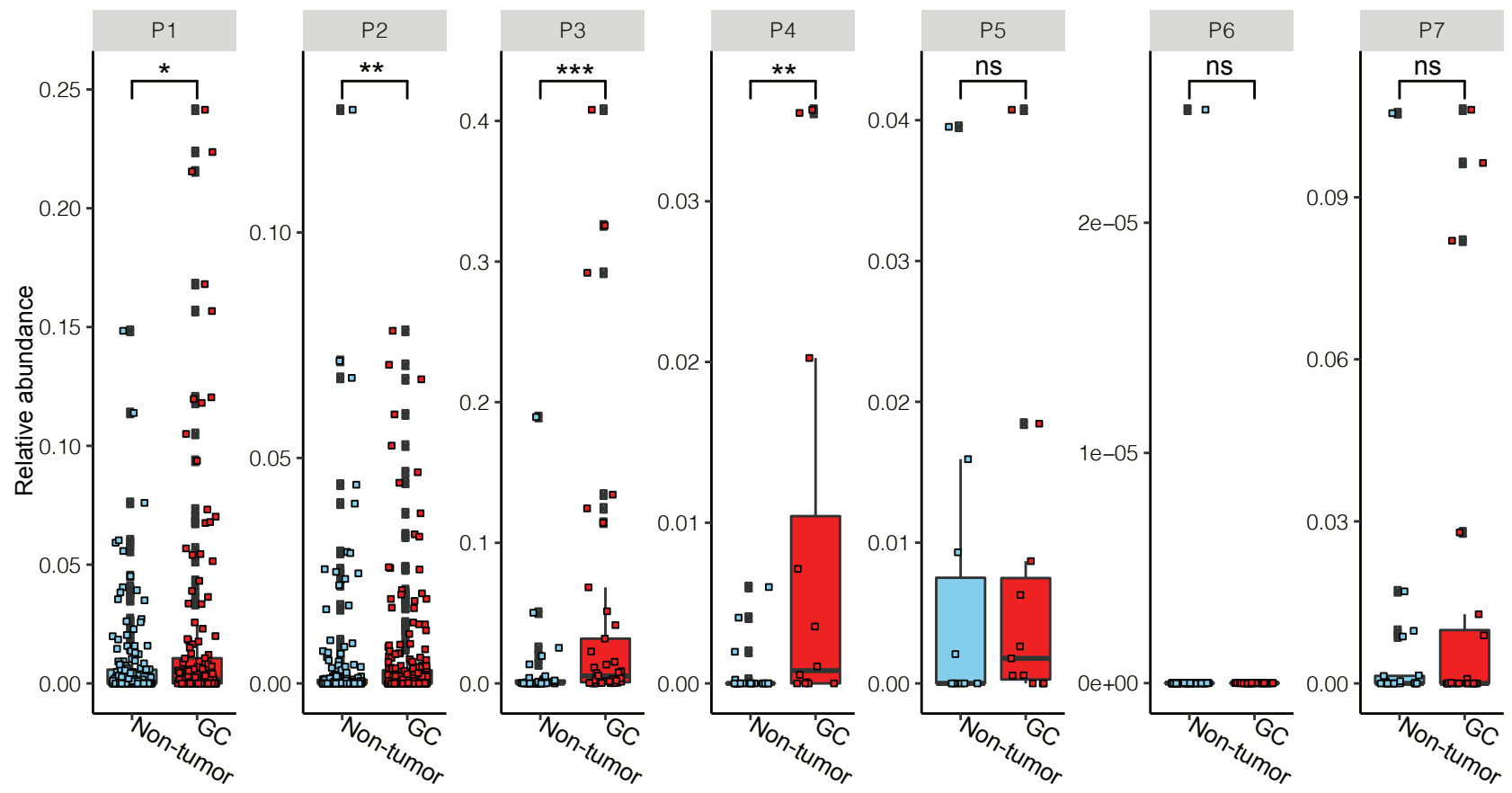

Supplement: qzaf132_Supplementary_Data [file qzaf132_supplementary_data.zip › Figure S5.pdf]

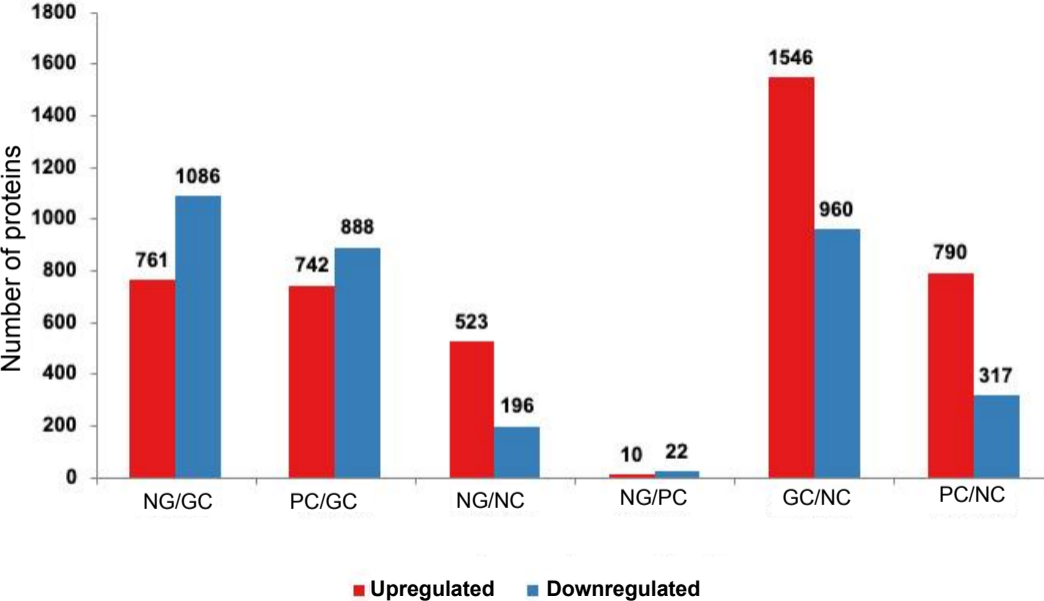

Supplement: qzaf132_Supplementary_Data [file qzaf132_supplementary_data.zip › Figure S6.pdf]

## TRYPTOPHAN METABOLISM

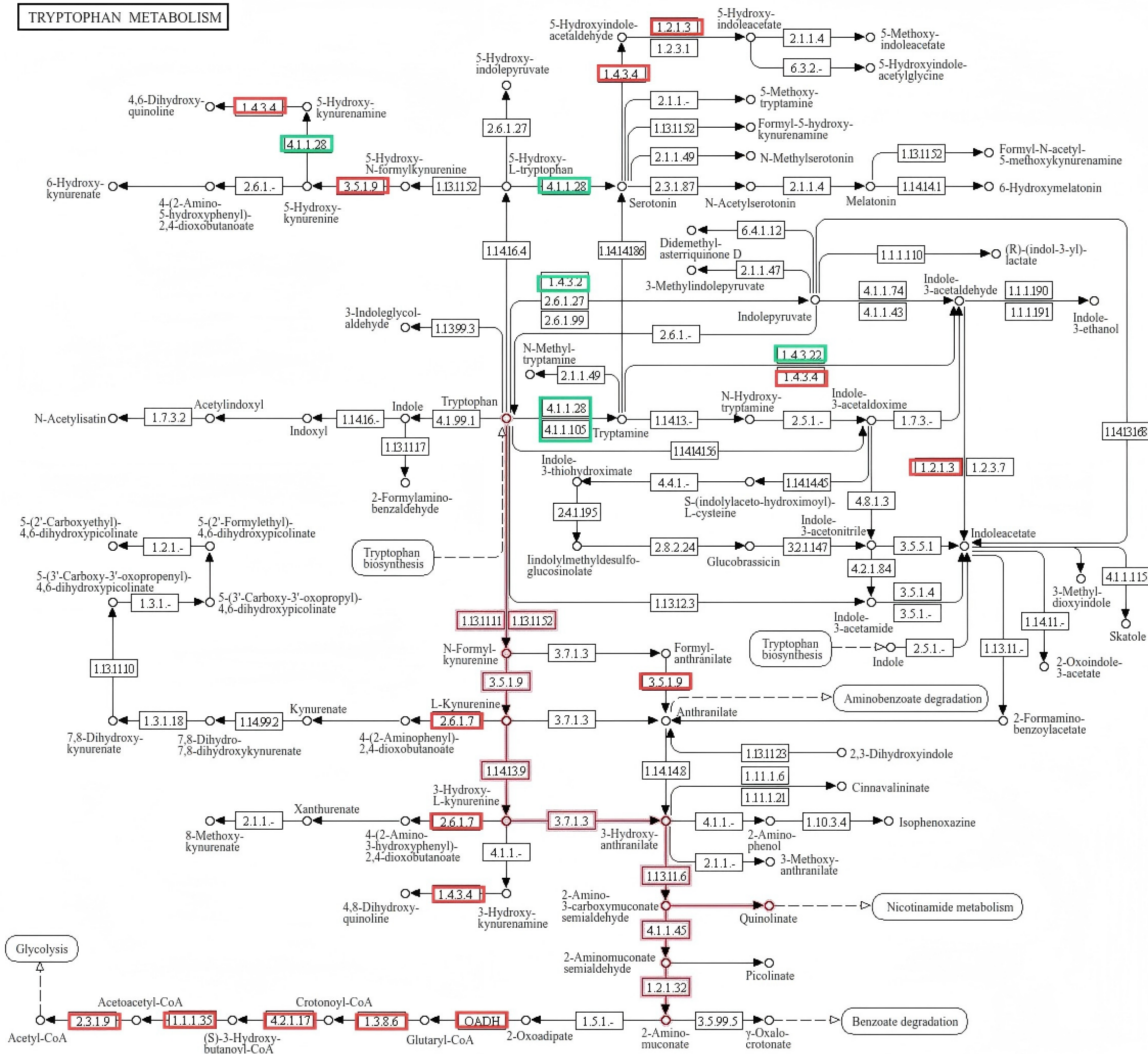

Supplement: qzaf132_Supplementary_Data [file qzaf132_supplementary_data.zip › Figure S7.pdf]

A

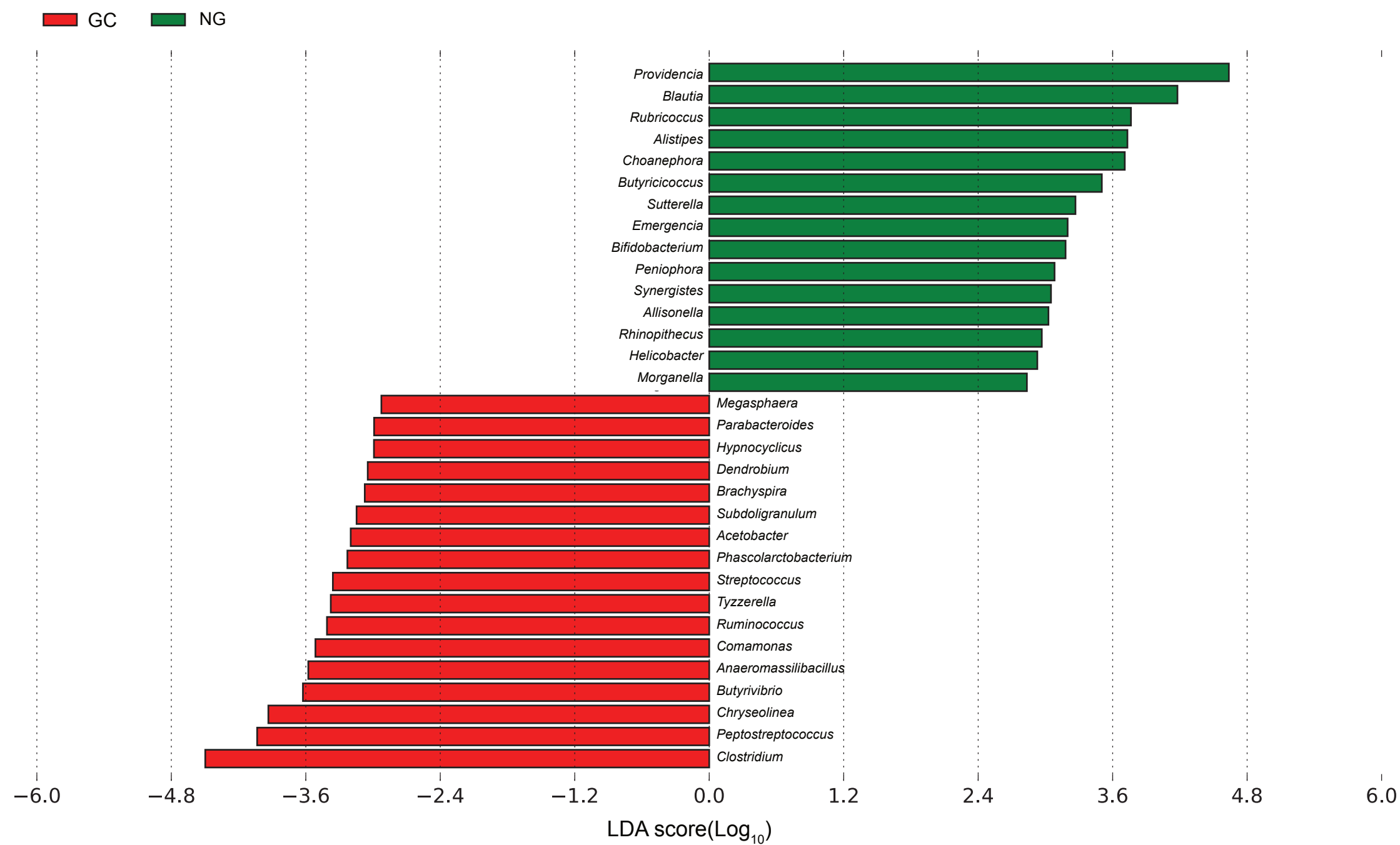

B

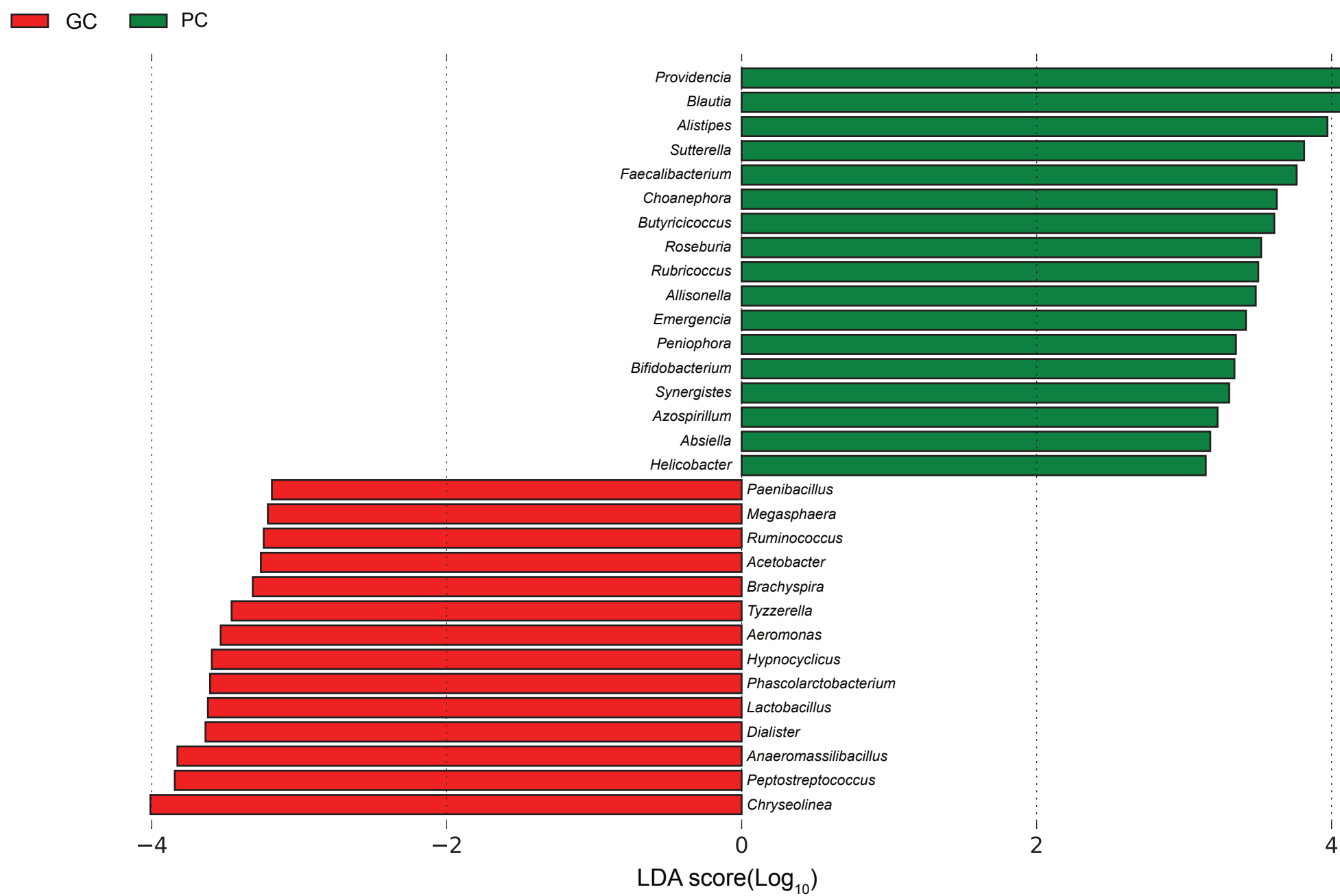

Supplement: qzaf132_Supplementary_Data [file qzaf132_supplementary_data.zip › Figure S8.pdf]
